# Acupuncture and moxibustion as adjunctive therapy for postoperative gastrointestinal dysfunction in gastric cancer: a systematic review and network meta-analysis

**DOI:** 10.3389/fmed.2024.1464749

**Published:** 2024-12-11

**Authors:** Yangxu Ou, Dezhi Lin, Xixiu Ni, Chengzhi Feng, Jing Rong, Xiaoyu Gao, Yang Yu, Xinrui Liu, Zhiyang Zhang, Wang Xiao, Zili Tang, Ling Zhao

**Affiliations:** ^1^Acupuncture and Tuina School, Chengdu University of Traditional Chinese Medicine, Chengdu, Sichuan, China; ^2^Hospital of Chengdu University of Traditional Chinese Medicine, Chengdu, China; ^3^Sichuan Clinical Medical Research Centre for Acupuncture and Moxibustion, Chengdu, China; ^4^Key Laboratory of Acupuncture for Senile Disease, Chengdu University of TCM, Ministry of Education, Chengdu, China

**Keywords:** postoperative gastrointestinal dysfunction, network meta-analysis, systematic review, acupuncture, moxibustion, time of first bowel sounds

## Abstract

**Objectives:**

This study aimed to evaluate and compare the efficacy and safety of different acupuncture and moxibustion techniques as adjunctive therapy in addressing Postoperative gastrointestinal dysfunction (PGD) associated with gastric cancer (GC).

**Methods:**

Eight medical databases were comprehensively searched for relevant randomized controlled trials (RCTs) as of October 2024. A network meta-analysis (NMA) was performed using frequency models, combining all available direct and indirect evidence from RCTs. Time of first bowel sounds (TFBS) was set as the primary outcome, and time to first defecation (TFD) and time to first flatus (TFF) were set as the secondary outcomes. All outcomes were ranked using surface under the cumulative ranking curve (SUCRA) probabilities to determine a hierarchy of treatments, and the probability that the intervention will be in one of the top ranks increases with a higher SUCRA value.

**Results:**

With 28 randomized controlled trials (RCTs) and 2,459 patients, 18 of which involved adjuvant acupuncture treatments. NMA based on SUCRA rankings showed that routine care (RC) with acupuncture (ACU), with acupressure (ACUP), with moxibustion (MOX) and acupoint injection (AI) were the top-ranked therapies for shortening TFBS and TFF in patients with GC compared with RC; additionally, RC + MOX + CUP and RC + MOX were the relatively best therapies for TFD. No serious adverse events were reported in the studies assessing the safety of adjunctive acupuncture therapy. Our study found that ST36, ST37, ST39, and PC6 were the most commonly used acupoints for adjuvant acupuncture treatments in treating PGD associated with GC.

**Conclusion:**

Acupuncture and moxibustion, when used as supplementary therapies, demonstrated efficacy and relative safety in managing PGD associated with GC. The recommended order for adjunctive acupuncture- and moxibustion-related therapies for PGD in patients with GC, in terms of conservativeness, is as follows: RC + ACU, RC + MOX + AI, RC + ACUP, RC + MOX + CUP and RC + MOX. Despite their inclusion, the overall methodological quality of the studies was poor, which need for further high-quality randomized controlled trials to support existing results.

**Systematic review registration:**

https://www.crd.york.ac.uk/PROSPERO.

## 1 Introduction

Gastric cancer (GC), primarily adenocarcinoma, is a malignant tumor affecting the gastric sinus, body, and cardio (1), ranking as the fifth most prevalent cancer globally and the third leading cause of cancer-related deaths (2). Surgical removal stands as the primary treatment for GC, where postoperative gastrointestinal dysfunction (PGD) emerges as the most common complication (3), marked by perioperative drug stimulation, intraoperative straining injury, direct injury from gastrointestinal surgery, and an excessive postoperative inflammatory response. This function results in varying degrees of gastrointestinal dysfunction, including the loss of bowel sounds, absence of defecation, and flatus (4), posing considerable risks such as intestinal adhesion, intestinal obstruction, and psychological comorbidities including anxiety and depression, alongside reduced quality of life (5, 6). Consequently, effective treatment and management of PGD following GC surgery, as well as efforts to improve patients’ quality of life and prognosis, have become pressing topics of research and clinical interest.

Conventional pharmacotherapy, featuring gastrin, erythromycin, domperidone, and cisapride (7). However, these Western medicine (WM) treatments exhibit limited efficacy and significant side effects, such as gastrointestinal inflammation, increased gastrointestinal burden, and cardiovascular complications from prolonged drug use (8–10). Current routine care (RC) for PGD in GC primarily involves fasting, gastrointestinal decompression, anti-infection, and nutritional support, aiming to maintain primary dietary status and prevent exacerbation; however, its impact on improving PGD in GC is limited. Consequently, complementary and alternative therapies, particularly RC-based adjuvant acupuncture-related treatments, have gained prominence in the management of PGD in GC.

Acupuncture, a traditional, non-pharmacological therapy, offers a green approach to addressing PGD in GC. Recent years have witnessed a surge in the use of various acupuncture- and moxibustion-related adjunctive therapies for gastrointestinal disorders, including irritable bowel syndrome (11, 12), gastroparesis (13), and constipation (14). Adjunctive acupuncture therapies such as auricular acupuncture (AA) (15), electroacupuncture (EA) (16), moxibustion (MOX) (17), point application therapy (PAT) (18), transcutaneous electrical acupoint stimulation (TEAS) (19), acupoint injection (AI) (20), cupping (CUP) combined with RC might offer advantages in treating PGD compared to RC alone or sham acupuncture (SA). A 2021 systematic review (21) highlighted the positive impact of RC combined with auricular acupressure on gastrointestinal function post-GC surgery. Another systematic review in 2022 (22) found that acupuncture, including warm acupuncture (WA), MOX, AA, and standard acupuncture (ACU), significantly improved the time to first defecation (TFD) and time to first bowel sounds (TFBS) in those with PGD among patients with GC. While the current evidence supports the consideration of adjunctive acupuncture- and moxibustion-related therapies in PGD management, a comprehensive understanding of the efficacy of various adjunctive acupuncture treatments for PGD in GC is lacking, posing challenges for clinicians in selecting the most effective approach.

This study employed a frequency model-based network meta-analysis (NMA) to compare the effects of diverse adjuvant acupuncture-related treatments for PGD in GC. The results of this study provide a basis for optimal acupuncture-related adjuvant therapy for PGD of GC and guide clinical practice. Additionally, the results aim to serve as a reference for optimizing postoperative care for patients experiencing PGD following GC surgery.

## 2 Methods

### 2.1 Registration

The study protocol was registered with the International Prospective Registry for Systematic Reviews (PROSPER) under CRD42022359145.

### 2.2 Search strategy

This study comprehensively searched eight databases from their inception to October 2024. Chinese databases included the Chinese Biomedical Literature Database, China National Knowledge Infrastructure, and the China Science and Technology Journal Database, Wan Fang database. English databases comprised PubMed, Embase, Web of Science, and The Cochrane Library. The inclusion criteria and search strategy were guided by the PRISMA protocol (23). Each database’s search terms were tailored, combining subject and free words. The search focused on [stomach neoplasm OR gastric cancer OR Gastric neoplasm OR Tumor of stomach] AND [Surgical Procedures, Operative OR operation OR Surgery OR post-operation OR Postoperative OR Post operation] AND [gastrointestinal dysfunction OR dysfunctional gastro intestine OR gastrointestinal function disturbance] AND [acupuncture therapy OR acupuncture OR moxibustion]. All included studies were randomized controlled trials (RCTs) involving human participants. The search process was independently conducted and verified by both authors. Moreover, a manual search of original articles and reviews was performed to augment the list of relevant studies. The search strategy is shown in Supplementary Tables S1–S8.

### 2.3 Eligibility criteria

(1) Patient(s): Those clinically diagnosed with GC (age > 18 years);
(2) Intervention(s): All needle or moxibustion therapies or both (AA, EA, SA, TEAS, PAT [point application therapy], acupressure [ACUP], MOX) combined with RC (RC comprises fasting, gastrointestinal decompression, anti-infective nutritional support, and emotional care);
(3) Control(s): RC, SA, sham moxibustion;
(4) Outcomes: TFBS, TFD, time to first flatus (TFF);
(5) Only RCTs were included.

### 2.4 Exclusion criteria

(1) Duplicate articles;
(2) Unavailable full-text studies;
(3) Non-PGD studies;
(4) Intervention group including oral herbal medicine;
(5) Outcome indicators for which data are either unavailable or cannot be analyzed on a consolidated basis.

### 2.5 Study selection and data extraction

Two authors (JR and XG) independently conducted screening and cross-checked eligible literature based on study type, population, measures, and outcome indicators for both intervention and control groups. Screening involved duplicate checking, initial screening of titles and abstracts, and thorough full-text reading. Moreover, two authors (YY and JR) independently conducted and cross-checked data extraction, encompassing author details, age, title, publication year, country, sex ratio, disease duration, interventions, randomization method, blinding, concealment, sample size, treatment duration, outcome metrics, and follow-up. Any discrepancies were resolved through discussion with another author (YO) for a final consensus. In cases of unclear or missing data, we contacted the corresponding study’s author via email.

### 2.6 Risk-of-bias assessment

Two authors (DL and YO) independently assessed the risk of bias assessment using the Cochrane Risk of Bias 2.0 (RoB 2.0) (24), and the five domains are: (1) bias arising from the randomization process; (2) bias due to deviations from the intended interventions; (3) bias due to missing outcome data; (4) bias in measurement of the outcome; and (5) bias in selection of the reported result (25). The results were categorized as “low risk of bias,” “some concerns,” or “high risk of bias.” Controversial situations were resolved through discussion with another author (XW).

### 2.7 Data analysis

The study integrated evidence from RCTs, treating outcome indicators such as TFBS, TFD, and TFF as continuous variables reported through mean differences (MDs) with 95% confidence intervals (CIs). A random-effects model was chosen to accommodate potential differences in the studies (26).

Data analysis and graphing were performed using STATA 15.1 (StataCorp, College Station, TX), employing the nodal method for quantitative analysis. Consistency between direct and indirect comparisons was tested, with *p* > 0.05 indicating a passed consistency test (27).

STATA 15.1 was further utilized for characterizing network graphs of various adjunctive acupuncture-related therapies, where each node represents a different intervention method. The connecting lines between the nodes represent direct comparisons among various intervention methods. The size of each node and the width of the connecting lines are proportional to the number of studies (28).

Recommendations for intervention therapies were primarily based on the surface of the cumulative ranking curve (SUCRA) value, represented as a P-score. The P-score, ranging from 0 to 1, indicates treatment effect magnitude, with 1 indicating the best and 0 the worst effect. While SUCRA or P scores aid in interpreting the effective percentage of relevant adjuvant acupuncture treatments, cautious interpretation is advised unless clinically meaningful variations between treatments are observed.

### 2.8 Publication bias

Funnel plots in the NMA visually confirmed the detection of publication bias. The symmetry of funnel plots for outcome metrics was observed to determine the probability of publication bias.

### 2.9 Quality of evidence

The GRADE method was utilized to determine the level of the confidence in the NMA estimations for efficacy outcomes (29). This hierarchical approach involves both direct and indirect estimates. Direct estimation was performed by starting with a high confidence level derived from included RCTs and downgrading it to moderate, low, or very low based on indirectness, heterogeneity, imprecision, risk of bias, or publication bias. Ratings for indirect estimates started at the lowest rating for two pairs of estimates, first-order cycles of indirect estimates. They were further downgraded for non-transmissibility or imprecision (studies differing in statistical methods or clinical inclusion criteria). Higher ratings, whether from direct or indirect sources, contributed to the overall quality of evidence in the NMA and were categorized as high, medium, low, or very low.

## 3 Results

### 3.1 Literature selection process

**Figure 1:**
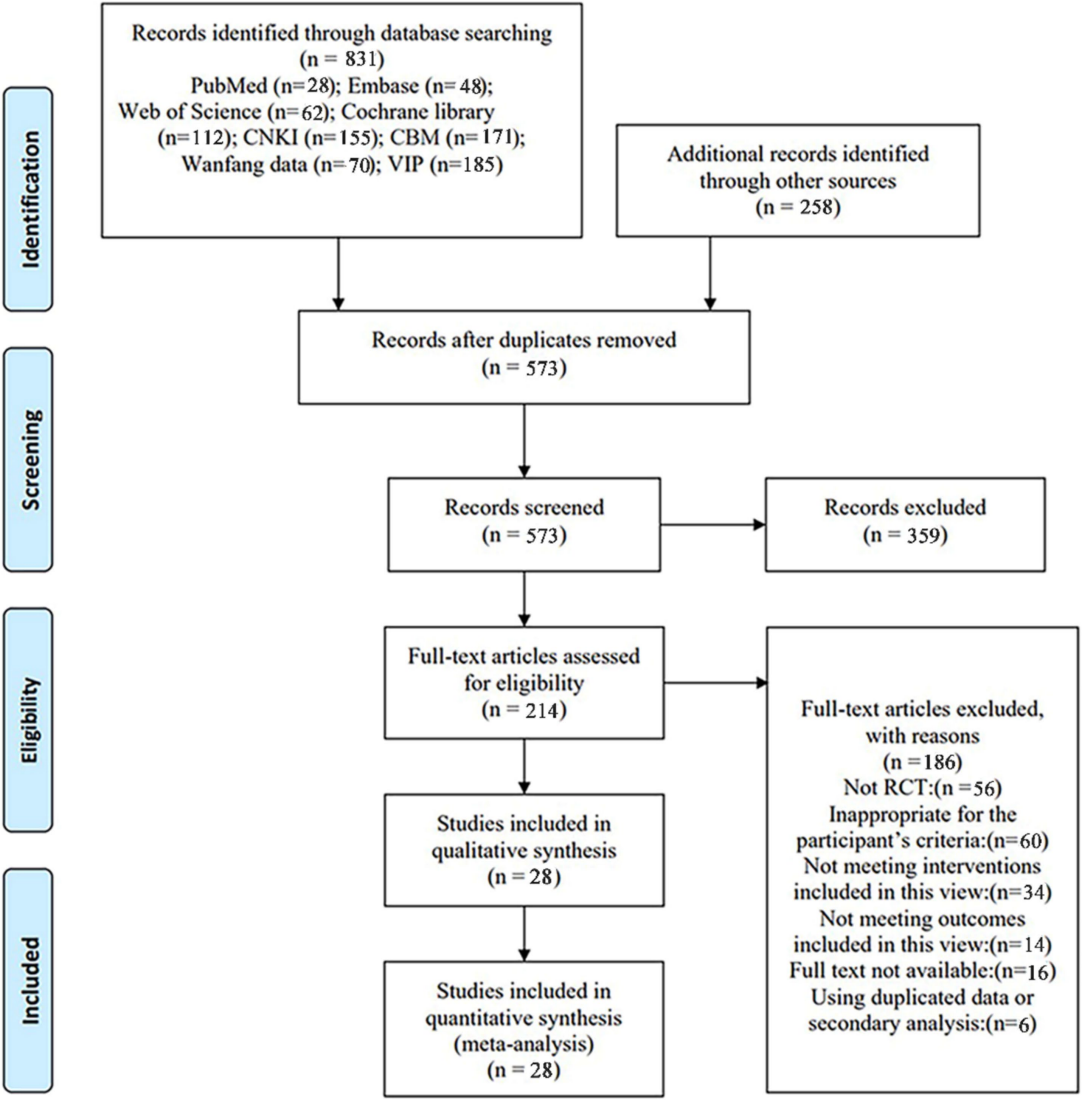
PRISMA flow chart. CNKI, China national knowledge infrastructure; VIP, China Science and Technology Journal Database; CBM, Chinese Biomedical Literature Database; RCT, randomized controlled trial.

A total of 831 studies were retrieved from the database for review. Initially, 258 duplicate studies were excluded, followed by the exclusion of 359 studies after reading the titles and abstracts from the remaining 573. Subsequently, 214 studies were excluded after a full-text reading of the remaining 186 (exclusions based on non-RCT, secondary analyses, unavailable full text, study population, study type, outcome metrics, and intervention method). Ultimately, 28 studies were included in this review, as illustrated in Figure 1 PRISMA flow chart.

### 3.2 Study characteristics

**Figure 2:**
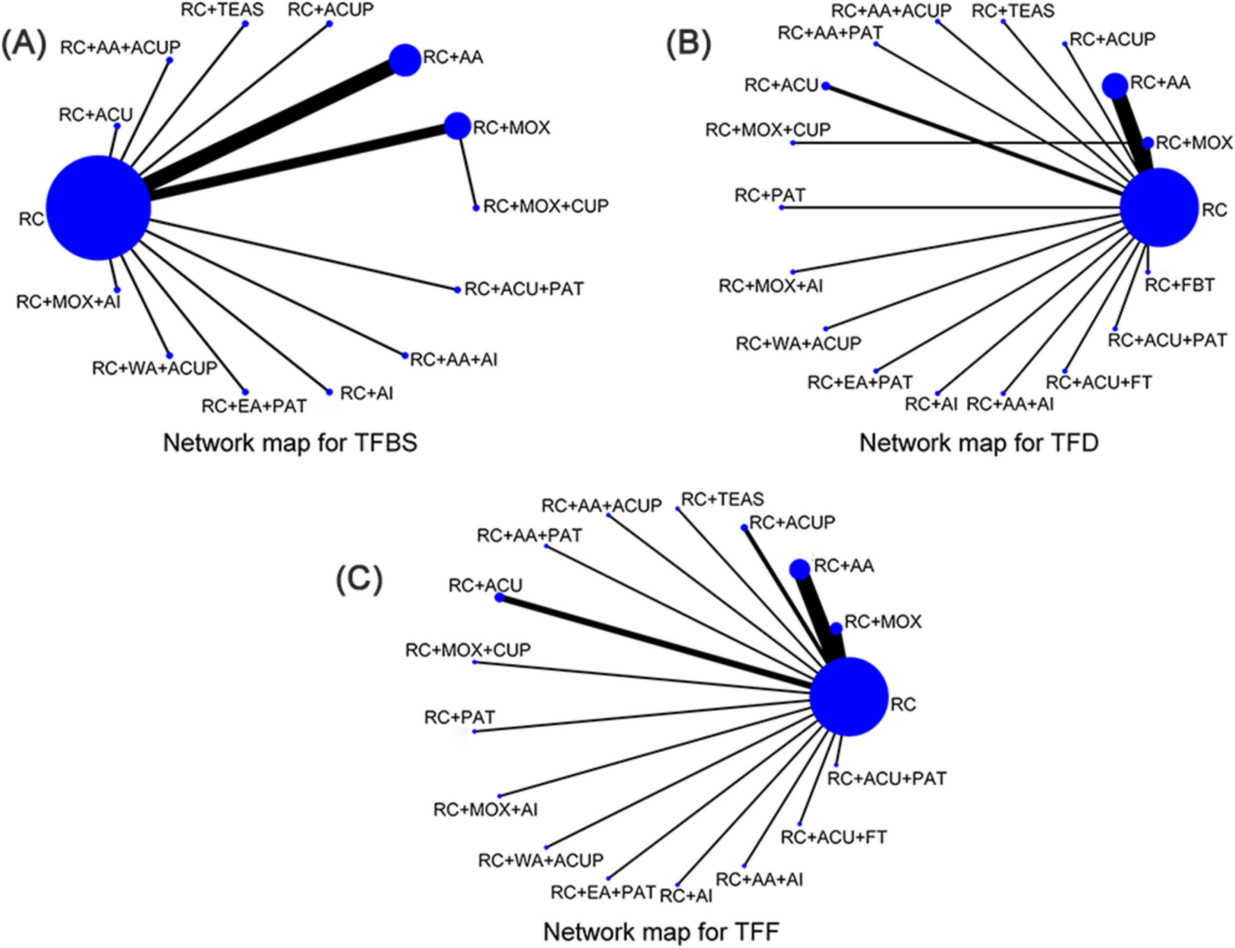
Network map for total score. **(A)** Network map for TFBS scores of gastric cancer. **(B)** Network map for TFD scores of gastric cancer. **(C)** Network map for TFF scores of gastric cancer. TFBS, Time of first bowel sounds; TFD, time to first defecation; TFF, Time to first flatus; RC, routine care; RC + MOX, routine care combined with moxibustion; RC + AA, routine care combined with auricular acupuncture; RC + ACUP, routine care combined with acupressure; RC + AA+ACUP, routine care combined with auricular acupuncture and acupressure; RC + ACU, routine care combined with acupuncture; RC + MOX + CUP, routine care combined with moxibustion and cupping; RC + MOX + AI, routine care combined with moxibustion and acupoint injection; RC + WA + ACUP, routine care combined with warming acupuncture and acupressure; RC + EA + PAT, routine care combined with electro-acupuncture and point application therapy; RC + AI, routine care combined with and acupoint injection; RC + AA+AI, routine care combined with auricular acupuncture and acupoint injection; RC + ACU+ FT, routine care combined with acupuncture and functional training; RC + ACU + PAT, routine care combined with acupuncture and point application therapy; RC + FBT, routine care combined with Foot bath therapy; RC + TEAS, routine care combined with transcutaneous electrical acupoint stimulation; RC + ACU + FT, routine care combined with acupuncture and functional training; RC + AA+PAT, routine care combined with auricular acupuncture and point application therapy; RC + PAT, routine care combined with point application therapy.

Through literature screening, 28 RCTs were included with 2,459 independent participants. Interventions in the 28 studies’ intervention groups mainly comprised RC combined with various acupressure-related treatments (RC + ACUP), point application therapy (RC + PAT), auricular acupuncture (RC + AA), acupuncture (RC + ACU), electroacupuncture (RC + EA), moxibustion (RC + MOX), electroacupuncture and point application therapy (RC + EA + PAT), and auricular acupuncture and acupressure (RC + AA+ACUP). The control group primarily involved RC, SA, RC + MOX, and RC combined with foot bath therapy (RC+ FBT). Key acupoints included ST36 (Zusanli), ST37 (Shangjuxu), LI4 (Hegu), ST39 (Xiajuxu), PC6 (Neiguan), ST25 (Tianshu), CV8 (Shenque), and auricular acupoints TF4 (Shenmen), AH6a (Jiaogan), CO4 (Wei), and AT4 (Pizhixia) (Table 1); The commonly used point combination is ST36-PC6. Network diagrams depicting primary and secondary outcome metrics are presented in Figure 2. The outcome network diagram for the primary outcome metric (TFBS) for this review is shown in Figure 2A, while the secondary outcome metrics (TFD, TFF) are shown in Figures 2B,C. The results of literature meeting study requirements were included in the systematic review, whereas some interventions were excluded from NMA analyses due to irrelevance or lack of available data.

**Table 1:** Basic features of included RCTs.

Study	Age (E/C)	Male (E/C)	Female (E/C)	Interventions (E/C)		Diagnostic criteria	Period of treatment (E/C)	Outcomes	Acupuncture points
				E	C				
Zhang 2014 (42)	53 ± 7.3/48 ± 9.6Y	12/28	10/30	RC + MOX	RC (① + ② + ③)	Clinical Diagnosis	Once daily (3D)/Once daily (3D)	1. TFBS; 2. TFF	ST36 *Zusanli* 足三里, ST37 *Shangjuxu*上巨虚, LI4 *Hegu* 合谷.
Ren 2016 (45)	63.27 ± 3.78/64.09 ± 3.74Y	18/14	19/13	RC + MOX	RC (① + ② + ③)	2013 Guidelines for the Standardized Treatment of Gastric Cancer	Once daily (1 W)/NR	1. TFBS; 2. TFF	ST36 *Zusanli* 足三里, CV8 *Shenque* 神阙.
Ding, 2021 (31)	46.00 ± 13.67/45.81 ± 13.82Y	21/11	18/14	RC + EA	RC (① + ② + ③ + SA)	Clinical Diagnosis	Once daily (5D)/Once daily (5D)	1. TFD; 2. TFF	GV20 *Baihui* 百会, ST36 *Zusanli* 足三里, PC6 *Neiguan* 内关, ST25 *Tianshu* 天枢.
Que 2021 (16)	65.38 ± 6.28/65.36 ± 6.18Y	21/19	22/18	RC + TEAS	RC (① + ② + ③ + ④)	2017 Experts on Early Stomach Cancer Screening Process in China Consensus opinion	Twice daily (NR)/NR	1. TFD; 2. TFBS; 3. TFF	CV12 *Zhongwan* 中脘, *Hegu* 合谷, ST25 *Tianshu* 天枢, ST37 *Shangjuxu*上巨虚
Sun, 2014 (46)	55.5 ± 5.7/54.5 ± 5.5Y	15/13	15/14	RC + AA	RC (① + ② + ③ + ④)	Clinical Diagnosis	Press 3–5 times a day, replacement once in two days (NR)/Press 3–5 times a day, replacement once in two days (NR)	1. TFD; 2. TFBS; 3. TFF	The following are auricular points: AT4 *Pizhixia* 皮质下, TF4 *Shenmen* 神门, AH6a *Jiaogan* 交感, CO7 *Dachang* 大肠, CO4 *Wei* 胃.
Nan, 2018 (51)	58.8 ± 4.7/59.3 ± 4.6Y	21/14	12/23	RC + AA	RC (① + ③)	Clinical Diagnosis	Press 3–5 times a day, replacement once in two days (4D)/8 times (4 W)	1. TFD; 2. TFF	The following are auricular points: AT4 *Pizhixia* 皮质下, TF4 *Shenmen* 神门, AH6a *Jiaogan* 交感, CO7 *Dachang* 大肠, CO4 *Wei* 胃.
Huang, 2014 (32)	63.02 ± 7.96/ 61.17 ± 7.47Y	52/48	60/40	RC + AA	RC (① + ② + ③)	Clinical Diagnosis	Press 3–5 times a day, replacement once in four days (1 W)/NR	1. TFD; 2. TFBS; 3. TFF	The following are auricular points: AT4 *Pizhixia* 皮质下, TF4 *Shenmen* 神门, AH6a *Jiaogan* 交感, CO7 *Dachang* 大肠, CO4 *Wei* 胃, CO13 *Pi* 脾.
Yuan, 2018 (50)	60.60 ± 11.00/58.60 ± 9. 70Y	25/23	27/21	RC + AA+PAT	RC (① + ② + ③)	2011 Guidelines for the Standardized Treatment of Gastric Cancer	Press 3–4 times a day, replacement once in four days (NR)/NR	1. TFD; 2. TFF	ST36 *Zusanli* 足三里, PC6 *Neiguan* 内关, SP6 *Sanyinjiao* 三阴交.
Ye, 2018 (41)	52.86 ± 3.52/52.11 ± 3.26Y	50/37	49/39	RC + AA	RC (① + ② + ③)	Clinical Diagnosis	Press 3–4 times a day, replacement once in four days (1 W)/1 W	1. TFD; 2. TFBS; 3. TFF	The following are auricular points: TF4 *Shenmen* 神门, AT4 *Pizhixia* 皮质下, CO13 *Pi* 脾, AH6a *Jiaogan* 交感, CO7 *Dachang* 大肠, CO4 *Wei* 胃.
Tan, 2017 (38)	73.5 ± 7.5岁/74.6岁 ± 6.2Y	20/14	12/22	RC + AA	RC (① + ② + ③)	2011 Guidelines for the Standardized Treatment of Gastric Cancer	Press 3–4 times a day, replacement once in three days (1 W)/1 W	1. TFD; 2. TFBS; 3. TFF	The following are auricular points: CO7 *Dachang* 大肠, CO4 *Wei* 胃, CO10 *Shen* 肾, CO17 *Sanjiao* 三焦.
Zou, 2021 (15)	50. 35 ± 10. 98/49. 67 ± 11. 12Y	28/22	29/21	RC + AA	RC (① + ② + ③)	Gastric Cancer Surgery	Press 3–4 times a day, replacement once in one days (2 W)/Once daily (2 W)	1. TFD; 2. TFBS; 3. TFF	The following are auricular points: CO4 *Wei* 胃, CO7 *Dachang* 大肠, CO13 *Pi* 脾, CO18 *Neifenmi* 内分泌.
Xu, 2017 (40)	48 ± 4.11/53 ± 4.47Y	45/10	46/7	RC + MOX	RC (① + ② + ③) + FBT	Clinical Diagnosis	Once daily (6D)/ Once daily (6D)	1. TFD; 2. TFBS; 3. TFF	/
Yang, 2022 (48)	51.79 ± 3.96/52.12 ± 4.27Y	28/12	26/14	RC + ACU	RC (① + ② + ③ + ④)	2013 Guidelines for the Standardized Treatment of Gastric Cancer	Once daily (10D)/NR.	1. TFD; 2. TFF.	ST36 *Zusanli* 足三里, SP9 *Yinglingquan*阴陵泉, SP6 *Sanyinjiao* 三阴交, ST37 *Shangjuxu*上巨虚, ST39 *Xiajuxu* 下巨虚
Liang, 2020 (34)	58.19 ± 8.92/57.47 ± 9.52Y	20/11	19/11	RC + MOX + CUP	RC (① + ② + ③) + MOX	2010 Diagnostic Criteria for Stomach Cancer	Once daily (5D)/Once daily (5D)	1. TFD; 2. TFBS; 3. TFF	ST36 *Zusanli* 足三里, ST36 *Zusanli* 足三里, ST37 *Shangjuxu*上巨虚, ST39 *Xiajuxu* 下巨虚.
Wu, 2020 (52)	62.40 ± 11.17/66.00 ± 2.12Y	13/2	11/4	RC + ACU	RC (① + ② + ③ + ④)	2010 Diagnostic Criteria for Stomach Cancer	NR/NR	1. TFD; 2.TFF.	ST37 *Shangjuxu*上巨虚, ST39 *Xiajuxu* 下巨虚.
Xie, 2020 (47)	52.83 ± 7.08/51.52 ± 6.31	51/33	49/35	RC + AA	RC (① + ② + ③ + ④)	Diagnostic criteria for radical gastric cancer surgery with Chinese and Western medicine	Press 3–4 times a day, replacement once in one days (1 W)/NR (1 W)	1. TFD; 2. TFBS; 3.TFF	The following are auricular points: CO4 *Wei* 胃, CO7 *Dachang* 大肠, AT4 *Pizhixia* 皮质下, AH6a *Jiaogan* 交感, CO13 *Pi* 脾, CO6 *Xiaochang* 小肠.
Mei, 2013 (44)	56.2 ± 4.5/57.0 ± 4. 8Y	38/12	33/9	RC + MOX + AI	RC (① + ② + ③ + ④)	Clinical Diagnosis	Once daily (1 W)/Once daily (1 W)	1. TFD; 2. TFBS; 3. TFF	ST36 *Zusanli* 足三里
Wu, 2021 (39)	56. 43 ± 10. 01/57. 38 ± 9.79Y	28/21	27/22	RC + WA + ACUP	RC (① + ② + ③)	Guiding Principles for Clinical Research on New Chinese Medicines	Once daily (1 W)/NR (1 W)	1. TFD; 2. TFBS; 3. TFF	PC6 *Neiguan* 内关, LI4 *Hegu* 合谷, ST36 *Zusanli* 足三里, ST36 *Zusanli* 足三里, PC6 *Neiguan* 内关, SP6 *Sanyinjiao* 三阴交, LR3 *Taichong* 太冲.
Lin, 2015 (35)	55.82 ± 13.19/57.57 ± 10.87Y	24/15	11/29	RC + EA + PAT	RC (① + ② + ③)	Clinical Diagnosis	Twice daily (NR)/NR	1. TFD; 2. TFBS; 3. TFF	ST36 *Zusanli* 足三里, CV8 *Shenque* 神阙.
Cui, 2014 (20)	60.6 (40–72)/62 (42–75) 46.2 ± 6.50Y	7/5	6/5	RC + AI	RC (① + ② + ③)	Clinical Diagnosis	Once daily (7D)/NR (7D)	1. TFD; 2. TFBS; 3. TFF	ST36 *Zusanli* 足三里
Zhang, 2022 (21)	69. 48 ± 8. 35/68. 60 ± 9. 06Y	31/15	33/16	RC+ AA+ ACUP	RC (① + ② + ③)	Clinical Diagnosis	Press 3 times a day, replacement once in three days (NR)/NR	1. TFD; 2. TFBS; 3. TFF	ST36 *Zusanli* 足三里, PC6 *Neiguan* 内关. The following are auricular points: TF4 *Shenmen* 神门, CO12 *Gan* 肝, CO4 *Wei* 胃, AH6a *Jiaogan* 交感.
Song, 2019 (37)	55.8 ± 6.6/55.7 ± 6.6Y	30/20	28/22	RC + ACUP	RC (① + ② + ③)	Clinical Diagnosis	Twice daily (5D)/NR (5D)	TFF; 2.TFBS	ST36 *Zusanli* 足三里, PC6 *Neiguan* 内关, LI4 *Hegu* 合谷
Li, 2016 (33)	65.2 ± 2.8/63.9 ± 3.4Y	34/7	35/10	RC+ AA+AI	RC (① + ② + ③)	Clinical Diagnosis	Once daily (NR)/Once daily (NR)	1. TFD; 2. TFBS; 3. TFF	ST36 *Zusanli* 足三里 The following are auricular points: TF4 *Shenmen* 神门, AT4 *Pizhixia* 皮质下, AH6a *Jiaogan* 交感, CO4 *Wei* 胃, CO7 *Dachang* 大肠, CO6 *Xiaochang* 小肠.
Qian, 2017 (36)	59 ± 10/60 ± 11Y	20/10	17/13	RC + ACU	RC (① + ② + ③)	Clinical Diagnosis	Once daily (1 W)/NR (1 W)	TFF; 2.TFBS.	ST36 *Zusanli* 足三里, ST37 *Shangjuxu*上巨虚, ST39 *Xiajuxu* 下巨虚.
Chen, 2017 (43)	44.9 ± 4.1/45.8 ± 3.9Y	28/19	24/18	RC + ACU + FT	RC (① + ④)	Clinical Diagnosis	Once daily (NR)/NR	1. TFD; 2.TFF	ST36 *Zusanli* 足三里, ST44 Neiting内庭 ST36 *Zusanli* 足三里, PC6 *Neiguan* 内关, LI4 *Hegu* 合谷, ST44 Neiting内庭.
Yuan, 2021 (49)	55.65 ± 7.49/55.69 ± 7.42Y	25/16	14/27	RC+ ACU+ PAT	RC (① + ② + ③)	Guidelines for the Treatment of Stomach Cancer	Twice daily (NR)/NR	1. TFD; 2. TFBS; 3. TFF	CV8 *Shenque* 神阙, LI4 *Hegu* 合谷, ST36 *Zusanli* 足三里, SP9 *Yinglingquan*阴陵泉.
Li, 2014 (18)	60.63 ± 7.81/58.91 ± 10.29Y	30/26	20/24	RC + PAT	RC (① + ② + ③)	Clinical Diagnosis	Once daily (NR)/NR	1. TFD; 2.TFF	CV8 *Shenque* 神阙
Wan-Ting Hsiung, 2015 (30)	60.54 ± 10.89/64.11 ± 15.60Y	20/6	20/8	RC + ACUP	RC (① + ② + ③)	Clinical Diagnosis	Once daily (3D)/NR (3D)	1. TFD; 2. TFF	ST36 *Zusanli* 足三里, PC6 *Neiguan* 内关.

E, experimental group; C, control group; D, day; W, week; M, month; Y, year; NR, not reported; N, none; WM, western medicine; Postoperative gastrointestinal dysfunction (PGD); RCTs, randomized controlled trials; AEs, adverse events; TFBS, Time of first bowel sounds; TFD, time to first defecation; TFF, Time to first flatus; RC, routine care; RC①, fasting; RC②, gastrointestinal decompression; RC③, anti-infective nutritional support; RC④, emotional care. RC + MOX, routine care combined with moxibustion; RC + EA, routine care combined with electro-acupuncture; RC + TEAS, routine care combined with transcutaneous electrical acupoint stimulation; RC + SA, routine care combined with sham acupuncture; RC + AA, routine care combined with auricular acupuncture; RC + AA + PAT, routine care combined with auricular acupuncture and point application therapy; RC + ACU, routine care combined with acupuncture; RC + MOX + CUP, routine care combined with moxibustion and cupping; RC + MOX + AI, routine care combined with moxibustion and acupoint injection; RC + WA + ACUP, routine care combined with warming acupuncture and acupressure; RC + EA + PAT, routine care combined with electro-acupuncture and point application therapy; RC + AI, routine care combined with and acupoint injection; RC + AA + ACUP, routine care combined with auricular acupuncture and acupressure; RC + ACUP, routine care combined with acupressure; RC + AA + AI, routine care combined with auricular acupuncture and acupoint injection; RC + ACU+ FT, routine care combined with acupuncture and functional training; RC + ACU + PAT, routine care combined with acupuncture and point application therapy; RC + FBT, routine care combined with Foot bath therapy; RC + PAT, routine care combined with point application therapy.

In the included studies, gender characteristics were reported for 2,459 patients, with 1,223 (51.2%) being women. The mean sample size was 90.3 (range, 60–200), and patient ages ranged from 28 to 80 years. All studies were conducted in China. Table 1 outlines the essential characteristics of the included literature.

### 3.3 Assessment of risk of bias

**Figure 3:**
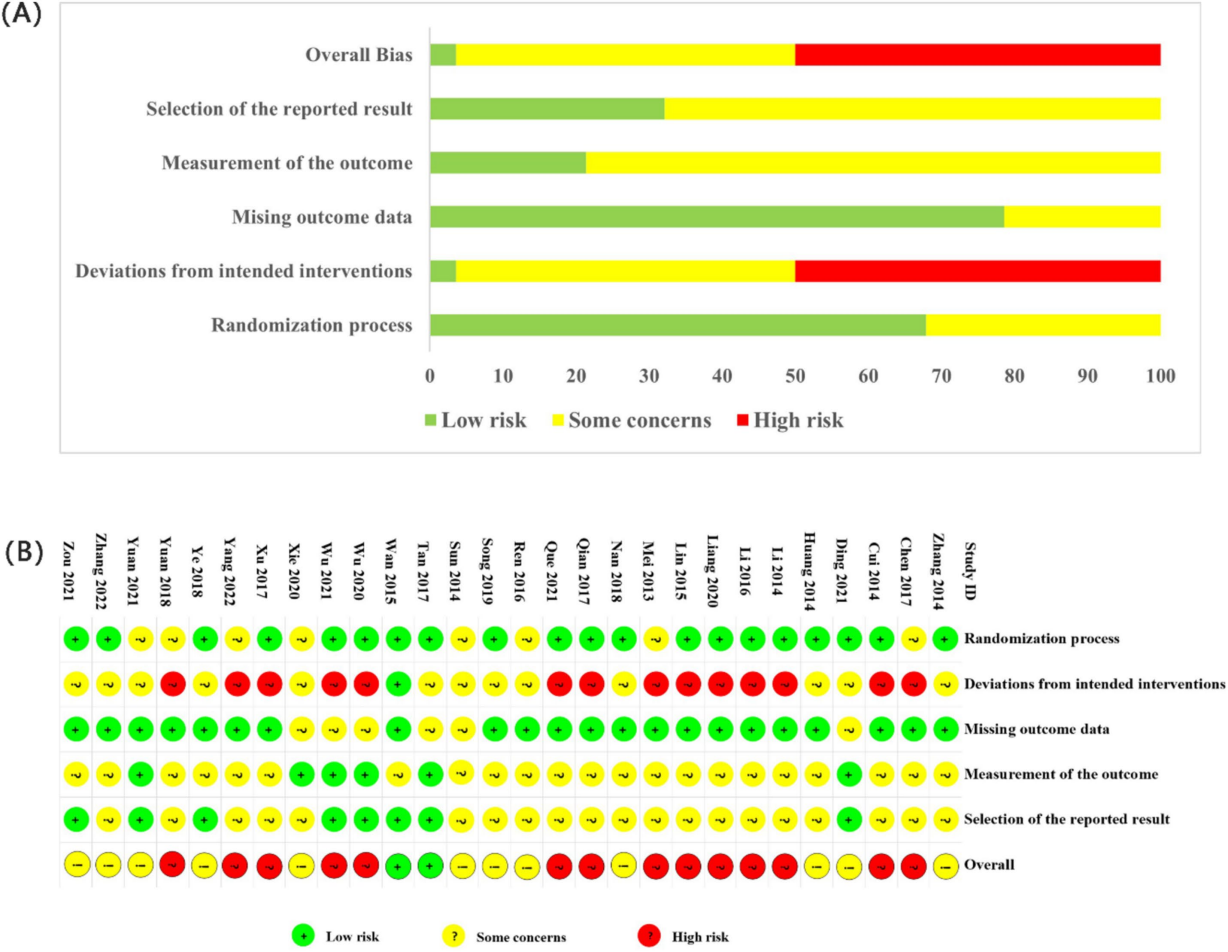
Bias risk assessment map. **(A)** Risk of bias graph. **(B)** Risk of bias summary.

The assessment of the risk of bias is illustrated in Figure 3. Across the five domains, 12 studies had “some concerns” as an overall rating, 14 had a “high risk of bias” and two was rated as of “low risk of bias.” Regarding randomization process, numerous studies employed random sequence generation methods with a low risk of bias (15–18, 30–42). Fifteen of these studies utilized random number tables (15–18, 32–42), one opted for random sampling (31), and another employed computer randomization (30). Studies that did not describe the randomization method were assigned an unclear risk of bias (20, 43–50). Concerning allocation concealment, one study utilized sealed opaque envelopes (30), and another employed central randomization (31). However, the remaining studies did not provide details about allocation concealment. Only the Wan-Ting Hsiung et al. study implemented blinding for researchers, outcome assessors, and participants (30). The other 11 studies did not specify their blinding methods and were assessed as having unclear risks (15, 32, 37, 38, 41, 42, 45–47, 50, 51). Furthermore, regarding bias due to deviations from the intended interventions, several studies were deemed high risk due to considerations related to the intervention’s nature (16, 18, 20, 33–36, 39, 40, 43, 44, 47–49, 52).

Concerning the blinding of outcome assessment, Ding et al. (31) and Wan-Ting Hsiung (30) et al. described blinding of outcome assessors and were rated as low risk. At the same time, the remaining studies did not mention blinding and were considered unclear. Regarding outcome data, 25 studies with complete data were regarded as low risk. For selective reporting, 16 studies had a low risk of bias, of which 8 (15, 30, 31, 38, 39, 41, 49, 52) received ethical approval; the remaining 20 were assessed as unclear. According to our study protocol, 21 studies that included specific statistical methods, baseline data, and exclusion criteria were considered low risk. The remaining studies (20, 38, 44, 46, 50, 51) were rated as unclear risk (20, 38, 44, 46, 50, 51). No dropouts were reported in the 28 studies.

### 3.4 Network meta-analysis

#### 3.4.1 Ranking of interventions

Ranking of interventions Using ranking probabilities and the surface under the cumulative ranking curves, the relative ranking of the various adjunctive acupuncture- and moxibustion-related interventions for each outcome was calculated. The cumulative probabilities of each treatment were expressed by a single value between 0 and 100%. The probability that the intervention will be in the top rank or in one of the top ranks increases with a higher percentage or SUCRA value (53).

#### 3.4.2 Results of NMA of TFBS

The review tested the consistency and inconsistency of all *p*-values for direct and indirect comparisons related to the TFBS. The results showed that the consistency model was acceptable (*p* = 0.62).

The comparison of efficacy based on adjuvant acupuncture-related therapies (marked in bold) is presented in Supplementary Table S9. RC + ACU (MD −23.9, 95%CI −35.39 to −12.41), RC + MOX + AI (MD −19.70, 95%CI −30.97 to −8.43), RC + ACUP (MD −19.20, 95%CI −30.22 to −8.18), RC + AA +AI (MD −18.70, 95%CI −29.90 to −7.50), RC + AI (MD −17.20, 95%CI −28.86 to −5.54), RC + WA + ACU (MD −13.37, 95%CI −24.40 to −2.34), and RC + ACU + PAT (MD −12.90, 95%CI −23.72 to −2.08) were all significantly different from RC. RC + ACU (MD −16.51, 95%CI −28.92 to −4.09) and RC + AI+MOX (MD −12.31, 95%CI −24.52 to −0.10) was all significantly different from RC + AA. RC + ACU (MD −20.57, 95%CI −36.37 to −4.77), RC + MOX + AI (MD −16.37, 95%CI −32.01 to −0.73), and RC + ACUP (MD −15.87, 95%CI −31.34 to −0.40) was significantly different from RC + AA+ACUP.

**Figure 4:**
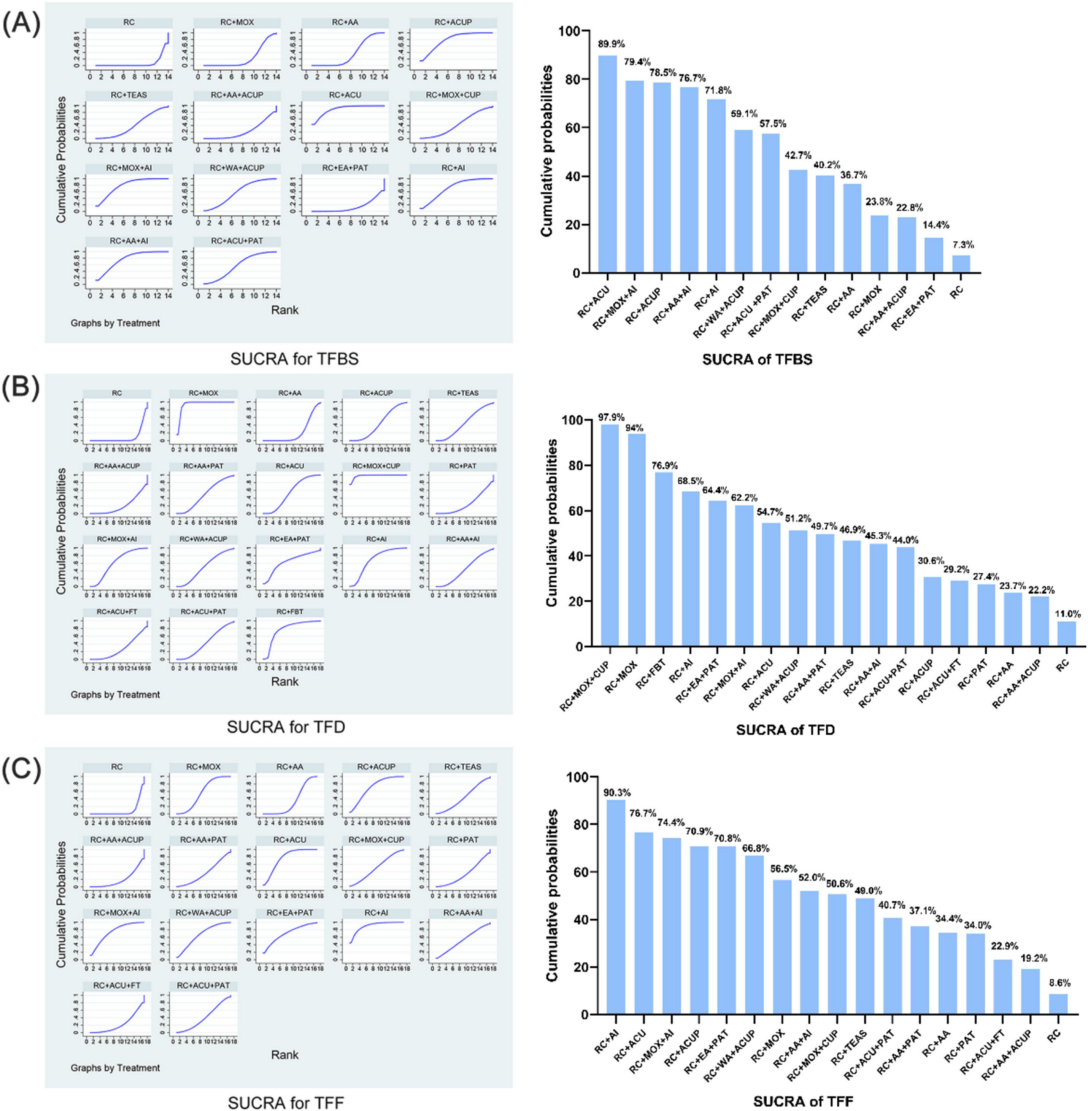
SUCRA for total score. **(A)** SUCRA for TFBS score of gastric cancer. **(B)** SUCRA for TFD score of gastric cancer. **(C)** SUCRA for TFF score of gastric cancer. TFBS, Time of first bowel sounds; TFD, time to first defecation; TFF, Time to first flatus; RC, routine care; RC + MOX, routine care combined with moxibustion; RC + AA, routine care combined with auricular acupuncture; RC + ACUP, routine care combined with acupressure; RC + AA+ACUP, routine care combined with auricular acupuncture and acupressure; RC + ACU, routine care combined with acupuncture; RC + MOX + CUP, routine care combined with moxibustion and cupping; RC + MOX + AI, routine care combined with moxibustion and acupoint injection; RC + WA + ACUP, routine care combined with warming acupuncture and acupressure; RC + EA + PAT, routine care combined with electro-acupuncture and point application therapy; RC + AI, routine care combined with and acupoint injection; RC + AA+AI, routine care combined with auricular acupuncture and acupoint injection; RC + ACU+ FT, routine care combined with acupuncture and functional training; RC + ACU + PAT, routine care combined with acupuncture and point application therapy; RC + FBT, routine care combined with Foot bath therapy; RC + TEAS, routine care combined with transcutaneous electrical acupoint stimulation; RC + ACU + FT, routine care combined with acupuncture and functional training; RC + AA+PAT, routine care combined with auricular acupuncture and point application therapy; RC + PAT, routine care combined with point application therapy.

The relative efficacy of the 14 interventions, including 21 trials, was estimated by NMA of the total TFBS score. The efficacy prioritization, based on SUCRA and mean rankings, is detailed in Figure 4A: RC + ACU (SUCRA 89.9%), RC + MOX + AI (79.4%), RC + ACUP (78.5%), RC + AA+AI (76.7%), RC + AI (71.8%), RC + WA + ACUP (59.1%), RC + ACU + PAT (57.5%), RC + MOX + CUP (42.7%), RC + TEAS (40.2%), RC + AA (36.7%), RC + MOX (23.8%), RC + AA+ACUP (22.8%), RC + EA + PAT (14.4%), and RC (7.3%).

#### 3.4.3 Results of NMA of TFD

The comparison of efficacy based on adjuvant acupuncture-related therapies (marked in bold) is presented in Supplementary Table S10. RC + MOX + CUP (MD −61.01, 95%CI −88.82, −33.21), RC + ACU (MD −16.80, 95%CI −30.86, −2.75), RC + MOX + AI (MD −20.80, 95%CI −39.83, −1.77), RC + AI (MD −24.60, 95%CI −44.43, −4.77), and RC + FBT (MD −33.46, 95%CI −62.22, −4.70) were all significantly different from RC. RC + ACU (MD −44.21, 95%CI −75.37, −13.06) and RC + AA (MD −56.60, 95%CI −85.34, −27.86) were all significantly different from RC + MOX + CUP.

The relative efficacy of the 18 interventions, including 21 trials, was estimated by NMA of the total TFD score. The efficacy prioritization, based on SUCRA and mean rankings in Figure 4B: RC + MOX + CUP (SUCRA 97.9%), RC + MOX (94%), RC + FBT (76.9%), RC + AI (68.5%), RC + EA + PAT (64.4%), RC + MOX + AI (62.2%), RC + ACU (54.7%), RC + WA + ACUP (51.2%), RC + AA+PAT (49.7%), RC + TEAS (46.9%), RC + AA+AI (45.3%), RC + ACU + PAT (44.0%), RC + ACUP (30.6%), RC + ACU + FT (29.2%), RC + PAT (27.4%), RC + AA (23.7%), RC + AA+ACUP (22.2%), and RC (11.0%).

#### 3.4.4 Results of NMA of TFF

The comparison of efficacy based on adjuvant acupuncture-related therapies, as highlighted in Supplementary Table S11, revealed that RC + AI (MD −28.00, 95%CI −43.22, −12.78), RC + ACU (MD −20.45, 95%CI −29.76, −11.13), RC + MOX + AI (MD −20.50, 95%CI −35.67, −5.33), RC + ACUP (MD −18.85, 95%CI −31.01, −6.69), RC + MOX (MD −14.30, −21.83, −6.76), and RC + AA (MD −8.34, 95%CI −14.21, −2.46) were all significantly different from RC. Moreover, RC + AI (MD −19.66, 95%CI −35.98, −3.35) substantially differed from RC + AA.

The relative efficacy of the 17 interventions, encompassing 21 trials, was assessed through NMA of the total TFF score. The prioritization of efficacy, based on SUCRA and mean rankings Figure 4C, is as follows: RC + AI (SUCRA 90.3%), RC + ACU (76.7%), RC + MOX + AI (74.4%), RC + ACUP (70.9%), RC + EA + PAT (70.8%), RC + WA + ACUP (66.8%), RC + MOX (56.5%), RC + AA+AI (52.0%), RC + MOX + CUP (50.6%), RC + TEAS (49.0%); RC + ACU + PAT (40.7%), RC + AA+PAT (37.1%), RC + AA (34.4%), RC + PAT (34.0%), RC + ACU + FT (22.9%), RC + AA+ACUP (19.2%), and RC (8.6%).

#### 3.4.5 Publication bias

**Figure 5:**
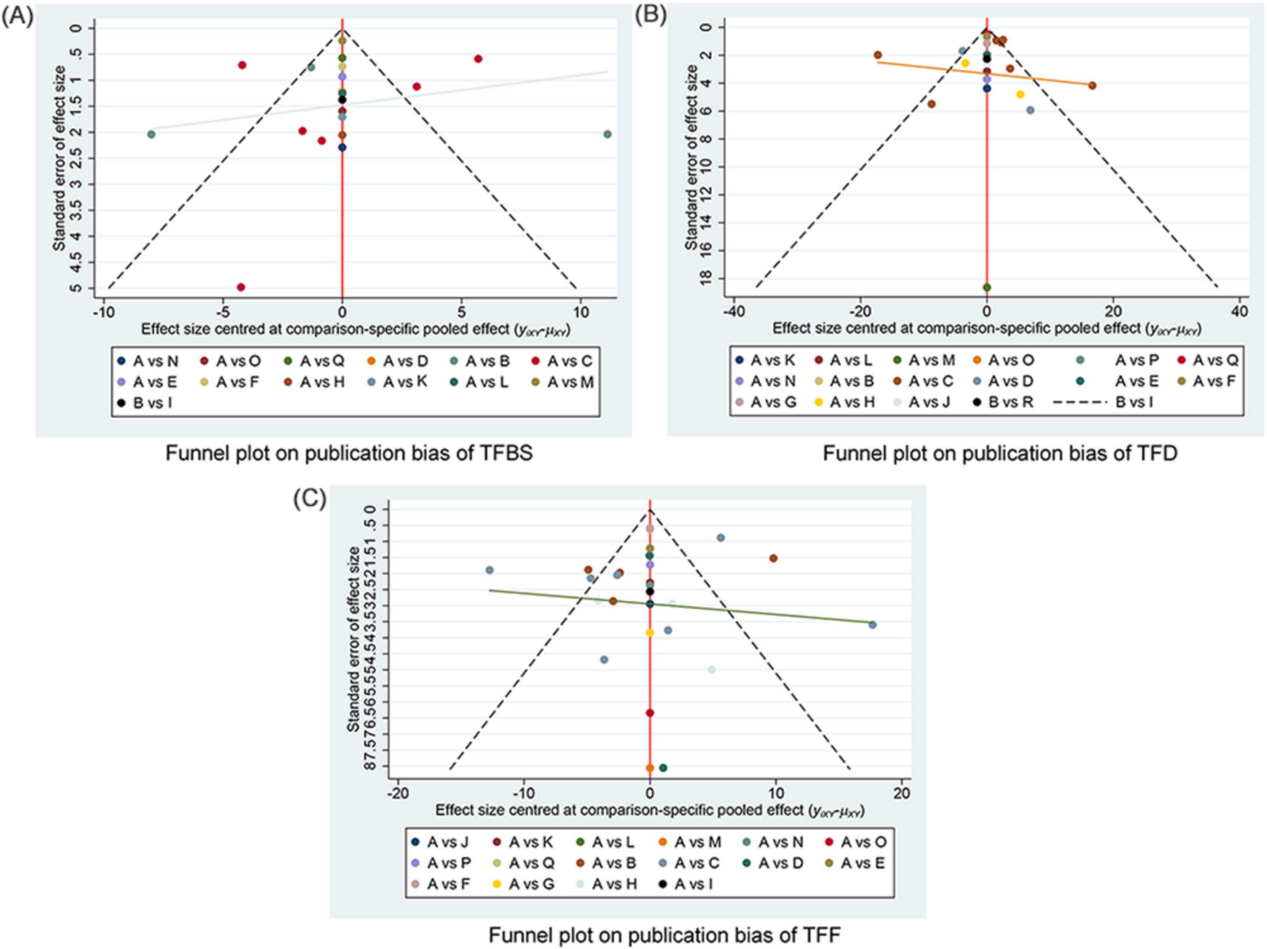
Funnel plot on publication bias. **(A)** Funnel plot publication bias of TFBS in gastric cancer. **(B)** Funnel plot publication bias of TFD in gastric cancer. **(C)** Funnel plot publication bias of TFF in gastric cancer. TFBS, Time of first bowel sounds; TFD, time to first defecation; TFF, Time to first flatus; A: RC, routine care, western medicine; B: RC + MOX, routine care combined with moxibustion; C: RC + AA, routine care combined with auricular acupuncture; D: RC + ACUP, routine care combined with acupressure; E: RC + TEAS, routine care combined with transcutaneous electrical acupoint stimulation; F: RC + AA+ACUP, routine care combined with auricular acupuncture and acupressure; G: RC + AA+PAT, routine care combined with auricular acupuncture and acupressure; H: RC + ACU, routine care combined with auricular acupuncture; I: RC + MOX + CUP, routine care combined with moxibustion and cupping; J: RC + PAT, routine care combined with point application therapy; K: RC + MOX + AI, routine care combined with moxibustion and acupoint injection; L: RC + WA + ACUP, routine care combined with warming acupuncture and acupressure; M: RC + EA + PAT, routine care combined with electro-acupuncture and point application therapy; N: RC + AI, routine care combined with and acupoint injection; O: RC + AA+AI, routine care combined with auricular acupuncture and acupoint injection; P: RC + ACU+ FT, routine care combined with acupuncture and functional training; Q: RC + ACU+ PAT, routine care combined with acupuncture and point application therapy; R: RC + AA+AI, routine care combined with auricular acupuncture and acupoint injection.

The funnel plots examining publication bias for primary and secondary outcomes are presented in Figure 5. Visual inspection of the plots did not reveal any significant publication bias.

### 3.5 Safety

#### 3.5.1 Adverse events

Among the six RCTs assessing AEs (30, 32, 34, 36, 41, 46), four studies on the RC + AA group reported AEs, such as nausea and vomiting, primarily attributed to postoperative reactions rather than acupuncture. The incidence remained lower than in the RC-only group (32, 36, 41, 46). One study on ACUP reported AEs (30), including one case of fatigue. Another study mentioned two patients transferred to the ICU due to infection exacerbation, likely associated with non-compliance with postoperative fasting (34). Therefore, these events are more likely related to surgery or patient compliance than acupuncture. Overall, acupuncture exhibited a low incidence of adverse events (AEs).

### 3.6 Quality of evidence

The overall quality of this review was assessed as follows: the NMA grading levels of the primary outcome indicator (TFBS) were mainly rated as three types: moderate, low, and very low (Supplementary Table S12). Moreover, the NMA grading levels of secondary outcome indicators (TFD, TFF) were predominantly rated as low and very low (Supplementary Tables S13, S14). The primary reasons for downgrading included imprecision in NMA results and the potential risk of bias.

## 4 Discussion

### 4.1 Summary of the main results

This study represents the initial attempt to assess the efficacy and safety of different acupuncture-assisted therapies in managing PGD in GC. It encompasses 28 studies with 2,459 patients.

Compared to RC, adjunctive acupuncture- and moxibustion-related therapies, such as RC + ACU, RC+ MOX + AI, RC + ACUP, RC+ AI, RC + MOX + CUP, and RC + MOX—demonstrated superior efficacy in alleviating PGD in GC. Specifically, NMA based on SUCRA rankings indicated that RC + ACU, RC + AI+MOX, RC + ACUP, and RC + AI were the top-ranked therapies for reducing the TFBS and TFF in patients with GC compared to RC. Additionally, we found that RC + MOX + CUP and RC + MOX had the highest probabilities of ranking first and second in treating TFD. There is a certain level of consistency among the various adjunctive acupuncture and moxibustion therapies for PGD in GC. However, it is essential to interpret these findings with caution due to the limited scope of interventions in this study. While few AEs were reported, apart from instances of nausea, vomiting, and fatigue associated with adjunctive acupuncture-related treatments, no severe AEs were documented.

### 4.2 Possible explanations for the present findings

The primary pathogenesis of postoperative PGD in patients with GC involves the inflammatory response and dysregulation of gastrointestinal hormonal activity triggered by surgical trauma, inhibiting gastrointestinal hormone secretion and the subsequent onset of PGD (4). Additionally, TFBS, TFF, and TFD may recover at various times following GC surgery, depending on the individual patient’s gastrointestinal health status.

#### 4.2.1 The role of acupuncture-related therapies in TFBS and TFF

In our review, acupuncture and moxibustion therapies, such as RC + ACU, RC + MOX + AI, and RC + ACUP, have emerged as more effective and relatively safe approaches for managing PGD, including TFBS and TFF in patients with GC. Previous studies have indicated that RC + ACU and RC + ACUP can significantly improve PGD in these patients, aligning with our findings (54, 55). TFBS indicates the onset of gastrointestinal activity, while TFF serves as a crucial marker for the recovery of intestinal function, both of which are important indicators of early gastrointestinal recovery after GC surgery. ACU involves mechanical stimulation of acupoints on the body’s surface, which promotes bowel sound production and facilitates the expulsion of intestinal gas by enhancing gastrointestinal peristalsis, stimulating hormone secretion, and reducing inflammation levels (56–58). Clinical studies have shown that applying ACU at ST37 and ST39 within 24 h post-GC surgery can restore abdominal bowel sound fluctuations to normal peristalsis, suggesting that ACU accelerates gastrointestinal recovery (51). Animal studies further indicate that ACU can elevate gastric motilin (MTL) levels, enhancing gastric motility and blood flow to the gastric mucosa, thereby promoting gastrointestinal function recovery (59). Additionally, ACU stimulation may activate 7nAchR, leading to changes in NF-κB activity and affecting inflammatory factor expression, which helps reduce intestinal inflammation and accelerates the production of bowel sounds and gas expulsion (60). However, the optimal frequency and timing of acupuncture for PGD in GC patients remain unclear and warrant further investigation.

Moreover, RC + MOX + AI is recommended for enhancing the recovery of TFBS and TFF following GC surgery. MOX provides warm stimulation to specific acupuncture points. Several clinical studies have demonstrated that MOX applied at the ST36, ST37, and LI4 acupoints based on RC can significantly shorten the recovery time for TFBS and TFF in patients with PGD related to GC, which is associated with improvements in local and systemic blood circulation, regulation of parasympathetic activity, and restoration of gastrointestinal rhythms (17, 45). Research indicates that gentle MOX at ST36 and ST39 in rats can downregulate the expression levels of substance P (SP) and vasoactive intestinal peptide (VIP) in the hypothalamus and colon tissues, highlighting the brain-gut interaction as a key neurological mechanism for enhancing gastrointestinal function through MOX (61). Neurotransmitters secreted by the autonomic nervous system can directly influence the enteric nervous system, facilitating rhythmic movements of the stomach and intestines. Studies have also shown that MOX at ST36 can increase the expression levels of gastric mucosa-associated factors nitric oxide (NO) and Bcl-2, emphasizing the nucleus tractus solitarius as the primary regulator of gastrointestinal reflexes (62). However, further clarification is needed regarding the temperature of MOX, the selection of MOX methods, and the timing of the procedure.

AI, which involves the injection of specific Chinese medicines such as Astragalus membranaceus and Western drugs like neostigmine into relevant acupoints, is used to prevent and treat diseases. A review study emphasized that AI can shorten the recovery time for PGD, aligning with our findings (63). Research indicates a close relationship between gastrin levels and gastrointestinal function, with neostigmine— a cholinesterase inhibitor— increasing gastrin content in the central nervous system, bloodstream, and local gastrointestinal tissues, thereby accelerating the recovery of TFBS and TFF (64). Astragalus membranaceus is renowned for its immune-enhancing properties, promoting tumor cell apoptosis and inhibiting tumor growth (65). AI therapy significantly prolongs the duration of drug action, enhances the therapeutic effects at acupoints, and combines the benefits of both drugs and acupuncture (20). It is believed to have longer-lasting effects compared to traditional acupuncture or simple intramuscular injections (66). Notably, studies have shown that the combination of RC + MOX + AI can promote the recovery of gastrointestinal function, improve tissue metabolism, and enhance immune function, proving to be more effective than RC or RC + MOX alone (44). However, further clarification is needed regarding the specific types and doses of drugs used in AI, as well as the mechanisms underlying the efficacy of RC + MOX + AI in treating PGD in GC patients.

Furthermore, RC + ACUP is recommended as a therapeutic option for PGD. ACUP involves stimulating acupoints on the body surface using fingers and palms, guided by the principles of meridian theory in traditional Chinese medicine. In Western medicine, ACUP is thought to engage vagal and parasympathetic efferent pathways, which release acetylcholine and adrenocorticotropic hormone, thereby inhibiting gastrointestinal inflammation and promoting recovery in PGD patients (67–69). ACUP enhances local blood circulation, nutrient and metabolite transport, and overall blood supply, reducing postoperative abdominal distension and effectively improving PGD indicators such as TFBS and TFF (70, 71). For patients with GC, ACUP can rapidly activate neural functions, enhance intestinal peristalsis, and ensure smooth intestinal transit (39, 42). The mechanical stimulation from abdominal acupressure accelerates peristalsis and promotes gastrointestinal flatus and defecation. Combining acupressure with acupuncture minimizes intestinal stimulation and reduces the burden on the liver and kidneys, improving patients’ tolerance to enteral nutrition (72, 73). However, further investigation is needed to clarify the specifics of acupressure, including its frequency, force, and duration.

#### 4.2.2 The role of acupuncture-related therapies in TFD

In our review, acupuncture and moxibustion-related therapies, such as RC + MOX + CUP and RC + MOX, demonstrated notable efficacy in treating PGD, particularly in relation to TFD in GC patients. TFD typically emerges last, often occurring several days post-surgery, and reflects further recovery of the intestinal tract. This process involves complex physiological functions, including the digestion and absorption of food as well as the formation and elimination of feces, making TFD an important marker of the patient’s return to a normal diet and gastrointestinal activity (74). As previously mentioned, RC + MOX plays a critical role in promoting peristalsis, enhancing the secretion of digestive juices, and regulating autonomic functions in the postoperative gastrointestinal tract. Interestingly, our findings indicate that the efficacy of RC + MOX + CUP surpasses that of both RC + MOX and RC alone. Cupping therapy employs specialized tools to create negative pressure on the skin’s surface, which promotes blood circulation, enhances tissue metabolism, and boosts immune function (75). Several studies have noted that combining moxibustion with cupping at acupoints ST36, ST37, and ST38 can significantly reduce the postoperative TFD and the time to resume eating compared to RC and RC + MOX (34, 76), aligning with the results of our study. Furthermore, RC + MOX + CUP was found to facilitate gastrointestinal motility and expedite the TFD process by modulating levels of gastrin (GAS), MTL, and cholecystokinin (CCK) (77). However, further investigation is needed to elucidate the operational details of cupping therapy, such as treatment frequency and retention time, as well as the mechanisms underlying the efficacy of RC + MOX + CUP.

In summary, we cautiously propose that adjunct therapies such as RC + ACU, RC + MOX + AI, and RC + ACUP may be beneficial in the early stages of PGD in patients with GC, potentially contributing to a reduction in TFBS and TFF. In the middle and late stages of recovery, RC + MOX + CUP and RC + MOX may prove more effective in accelerating TFD. In future clinical practice, the selection of postoperative care and complementary acupuncture therapies should be tailored to the patient’s postoperative timeline and gastrointestinal function status, as this may enhance overall recovery. We hope that higher-quality studies will be conducted in the future to further validate our findings and to elucidate the mechanisms underlying the efficacy of acupuncture in treating PGD in patients with GC.

#### 4.2.3 Explanations related to the selection of acupuncture points

This study identified ST36, ST37, ST39, and PC6 as the most frequently used acupoints for treating PGD in GC. The emerging “gastrointestinal-neuro-endocrine-immune network regulation” theory has spotlighted acupuncture as a mechanism for gastrointestinal function regulation. Clinical and experimental evidence supports postoperative acupuncture’s role in modulating brain-gut peptide (BGP) expression, promoting gastrin secretion, enhancing gastrointestinal motility, and expediting postoperative recovery, reducing complications (72, 78). Abdominal acupoint stimulation inhibits motility in specific gastrointestinal segments by increasing the activity of sympathetic efferent fibers. In contrast, extremity acupoint stimulation promotes gastrointestinal motility by stimulating the activity of vagal efferent fibers (79, 80). Huang et al. found that EA on ST37 modulates gastrointestinal motility by activating the solitary tract’s nucleus and the vagus nerve’s dorsal motor nucleus, indicating a neural pathway-based modulatory effect (81). The auricular region represents the only area on the body surface where vagus nerve distributions are concentrated. The frequently used auricular acupoints in this study included CO4 (Wei), AH6a (Jiaogan), AT4 (Pizhixia), and TF4 (Shenmen). Stimulation of AT4 (Pizhixia) and TF4 (Shenmen) primarily affects the regulation of the cerebral cortex, while stimulation of CO4 (Wei) and AH6a (Jiaogan) can influence the vagus nerve, thereby promoting gastrointestinal peristalsis and the secretion of digestive juices, ultimately facilitating the recovery of gastrointestinal function (51, 82).

Among acupuncture point combinations, the pairing of ST36-PC6 is deemed the most stable in acupuncture prescriptions for PGD in GC. ST36 stimulation reduces gastrointestinal tract inflammation, improves blood flow, and regulates gastric motility production (83, 84). Simultaneously, PC6 regulates endocrine function, primarily adrenaline and antidiuretic hormone, reducing gastric acid secretion and regulating gastrointestinal function (85). Acupuncture at PC6-ST36 addresses the inflammatory state of the gastrointestinal tract, regulates gastrointestinal hormone levels, and facilitates the recovery of gastrointestinal function in patients with GC (86, 87). In summary, acupuncture primarily modulates PGD by regulating the gastrointestinal “neural-endocrine-immune” pathway.

### 4.3 Limitations

#### 4.3.1 Quality evidence of included studies

For several reasons, all outcome indicators (TFBS, TFD, TFF) in this review exhibited low-quality evidence for most studies, as per GRADE scores. Firstly, the randomization methods in the enrolled studies were vaguely described: among the 28 included RCTs, nine did not describe the randomization process (20, 43–50). Secondly, allocation concealment was mainly absent in the included studies, with only one RCT providing information on allocation concealment (30). Thirdly, there was a lack of standardization in the statistical methods, baseline data, and diagnostic criteria used across the included studies, introducing heterogeneity that needs consideration when interpreting the review results for potential bias. In summary, the quality of evidence for the results of the included studies was generally low. The biases mentioned above could contribute to false-positive results, and we should approach the findings cautiously.

#### 4.3.2 Inconsistent interventions

Due to the limited literature included in this review, it was impossible to encompass all interventions. Some interventions were represented by only one or two studies, introducing limitations to the results. Furthermore, the potential bias of the included studies should be noted concerning the duration, frequency, acupuncture point selection, number of points, needle retention duration, and needle depth, which may have varied. Therefore, a cautious interpretation of the final ranking results of this review is warranted.

#### 4.3.3 Limited outcomes

This review had limited coverage of outcome indicators, hindering a comprehensive evaluation of the differences in the efficacy of adjuvant acupuncture for treating patients with PGD in GC. Only six studies reported AEs, and none addressed follow-up. Consequently, the safety and long-term effects of adjuvant acupuncture-related treatments for PGD in GC warrant more in-depth and comprehensive investigations.

### 4.4 Comparison with other reviews and strengths and limitations of this review

Two previous meta-analyses have assessed the efficacy of acupuncture for treating PGD in patients with GC. The findings indicated that acupuncture may facilitate the recovery of PGD; however, the comparisons between various acupuncture interventions remain unclear (22, 88). Another meta-analysis demonstrated that, compared to RC, acupuncture can reduce TFF and TFBS following cancer surgery. Nonetheless, the safety evaluation of different interventions for PGD in the context of cancer surgery remains contentious (54). A recently published review examining various acupuncture therapies for gastrointestinal dysfunction post-gastric and colorectal cancer indicated that MOX, WA, ACUP, and ACUP were superior to RC. However, a systematic network meta-analysis comparing the efficacy of these different interventions is currently lacking (55). In summary, prior reviews and meta-analyses have preliminarily suggested that acupuncture can aid in the recovery of PGD in patients with GC. However, there is a notable absence of systematic and comprehensive evaluations of the efficacy and safety of different therapies, which represents a significant strength of the present study.

NMA is as an excellent tool for selecting the best therapy among multiple options. Given the limitations of available treatments for PGD in patients with GC, complementary alternative therapies offer additional options; however, few studies have comprehensively evaluated the efficacy of different adjuvant acupuncture therapies. The strength of this review lies in comparing the efficacy and safety of various adjuvant acupuncture therapies for PGD in GC using the NMA method, providing a valuable reference for patients and clinicians in selecting optimal adjuvant acupuncture.

This study has several limitations, including the following points. Firstly, the search language of this study included only Chinese and English, which could introduce potential bias due to language restrictions. Second, the effect of adjuvant acupuncture on PGD in patients with GC remains unclear as none of the included literature reported on follow-up. Third, the quality of the included literature was generally rated as low, possibly due to poor study design and the limited number of included studies. Fourth, the severity of PGD after GC was missing from the included studies, so the results of the efficacy of acupuncture of different severity of PGD are unclear.

Upon analysis and summary of this review, it becomes apparent that few studies have focused on observing the course and follow-up of PGD in GC. Therefore, more high-quality studies are encouraged to investigate the effects of adjuvant acupuncture on the treatment of PGD in GC further, covering aspects such as durability, safety, and efficacy.

## 5 Conclusion

Acupuncture and moxibustion emerged as effective and well-tolerated adjuvant therapies for managing PGD in GC. In a relatively conservative hierarchy, the recommended order of adjunctive acupuncture- and moxibustion-related treatments for PGD in GC includes RC + ACU, RC + MOX + AI, RC + ACUP, RC + MOX + CUP and RC + MOX. Nevertheless, the methodological quality of the included studies was generally poor, which need further well-designed, high-quality, large-scale, multicenter RCTs studies to substantiate our results.

## Data Availability

The original contributions presented in the study are included in the article/Supplementary material, further inquiries can be directed to the corresponding author.

## References

[1] Yakirevich E Resnick MB. Pathology of gastric cancer and its precursor lesions. Gastroenterol Clin N Am. (2013) 42:261–84. doi: 10.1016/j.gtc.2013.01.004 23639640

[2] Bray F Ferlay J Soerjomataram I Siegel RL Torre LA Jemal A. Global cancer statistics 2018: globocan estimates of incidence and mortality worldwide for 36 cancers in 185 countries. CA Cancer J Clin. (2018) 68:394–424. doi: 10.3322/caac.21492, PMID: 30207593

[3] Feng RM Zong YN Cao SM Xu RH. Current cancer situation in China: good or bad news from the 2018 global cancer statistics? Cancer Commun (Lond). (2019) 39:22. doi: 10.1186/s40880-019-0368-6, PMID: 31030667 PMC6487510

[4] Kovoor JG Stretton B Jacobsen J Gupta AK Ovenden CD Hewitt JN . Gastrointestinal recovery after surgery: protocol for a systematic review. BMJ Open. (2021) 11:e54704:e054704. doi: 10.1136/bmjopen-2021-054704, PMID: 34645666 PMC8515468

[5] Wang J Li Y Qi Y. Effect of glutamine-enriched nutritional support on intestinal mucosal barrier function, mmp-2, mmp-9 and immune function in patients with advanced gastric cancer during perioperative chemotherapy. Oncol Lett. (2017) 14:3606–10. doi: 10.3892/ol.2017.6612, PMID: 28927119 PMC5588077

[6] Wang LL Liang JH Ruan XX Jin CH. Clinical study of hot ironing with moxa salt packet combined with auricular pressure beans for postoperative chemotherapy of gastric cancer. New Chinese Medicine. (2021) 53:147–51. doi: 10.13457/j.cnki.jncm.2021.02.037

[7] Oh SY Lee HJ Yang HK. Pylorus-preserving gastrectomy for gastric cancer. J Gastric Cancer. (2016) 16:63–71. doi: 10.5230/jgc.2016.16.2.63, PMID: 27433390 PMC4944004

[8] Masuy I Van Oudenhove L Tack J. Review article: treatment options for functional dyspepsia. Aliment Pharmacol Ther. (2019) 49:1134–72. doi: 10.1111/apt.15191 30924176

[9] Sakamoto Y Kato S Sekino Y Sakai E Uchiyama T Iida H . Effects of domperidone on gastric emptying: a crossover study using a continuous real-time 13c breath test (breathid system). Hepato-Gastroenterology. (2011) 58:637–41. PMID: 21661445

[10] Tougas G Earnest DL Chen Y Vanderkoy C Rojavin M. Omeprazole delays gastric emptying in healthy volunteers: an effect prevented by tegaserod. Aliment Pharmacol Ther. (2005) 22:59–65. doi: 10.1111/j.1365-2036.2005.02528.x, PMID: 15963081

[11] Ma XP Hong J An CP Zhang D Huang Y Wu HG . Acupuncture-moxibustion in treating irritable bowel syndrome: how does it work? World J Gastroenterol. (2014) 20:6044–54. doi: 10.3748/wjg.v20.i20.6044 24876727 PMC4033444

[12] Pei L Geng H Guo J Yang G Wang L Shen R . Effect of acupuncture in patients with irritable bowel syndrome: a randomized controlled trial. Mayo Clin Proc. (2020) 95:1671–83. doi: 10.1016/j.mayocp.2020.01.042 32499125

[13] Wang CP Kao CH Chen WK Lo WY Hsieh CL. A single-blinded, randomized pilot study evaluating effects of electroacupuncture in diabetic patients with symptoms suggestive of gastroparesis. J Altern Complement Med. (2008) 14:833–9. doi: 10.1089/acm.2008.0107, PMID: 18721079

[14] Liu Z Yan S Wu J He L Li N Dong G . Acupuncture for chronic severe functional constipation: a randomized trial. Ann Intern Med. (2016) 165:761–9. doi: 10.7326/M15-3118 27618593

[15] Zou SD. Effect of auricular pressure bean nursing on recovery time of gastrointestinal function and clinical symptom scores of patients undergoing laparoscopic radical gastric cancer surgery. Integrat Nurs Chinese Western Med. (2021) 7:41.

[16] Que HL. Effects of acupoint stimulation with electroacupuncture instrument combined with affective care on self-efficacy, gastrointestinal function and quality of life of perioperative patients with gastric tumours. New Chinese Med. (2021) 53:188–91. doi: 10.13457/j.cnki.jncm.2021.24.050

[17] Zhang YW Li AY Ye JY. The efficacy of moxibustion on the recovery of intestinal peristalsis after gastric cancer surgery. Nurs Rehabil. (2014) 13:795–6.

[18] Li JY Huang WJ Wu YH. Observation on the effect of traditional Chinese medicine applying Shenque acupoints to promote the recovery of gastrointestinal function in postoperative patients with gastric cancer. J Nurs. (2014) 21:60–1.

[19] Jiang T Li J Meng L Wang J Zhang H Liu M. Effects of transcutaneous electrical acupoint stimulation on gastrointestinal dysfunction after gastrointestinal surgery: a meta-analysis. Complement Ther Med. (2023) 73:102938. doi: 10.1016/j.ctim.2023.102938 36842636

[20] Cui R Zhang HY Ren QS Xu S. Therapeutic efficacy of neostigmine foot-sanli acupoint injection in the treatment of gastrointestinal dysfunction after gastric cancer surgery. J Tradit Chin Med. (2014) 20:63–4. doi: 10.13862/j.cnki.cn43-1446/r.2014.10.022

[21] Zhang R Guo LQ Tang YY Zhang RJ Wang ZP Wang J. Meta-analysis of the effect of auricular acupressure on the recovery of gastrointestinal function in gastric cancer patients after surgery. Evid Based Nurs. (2021) 2021:1–12. doi: 10.1155/2021/3996101

[22] Li HY Chen Y Hu ZY Chen P Li RL Jiang JW. Meta-analysis of the efficacy of acupuncture in treating gastrointestinal dysfunction after gastric cancer surgery. Zhongguo Zhen Jiu. (2022) 42:595–602. doi: 10.13703/j.0255-2930.20210214-0003 35543956

[23] Page MJ McKenzie JE Bossuyt PM Boutron I Hoffmann TC Mulrow CD . The prisma 2020 statement: an updated guideline for reporting systematic reviews. BMJ. (2021) 372:n71. doi: 10.1136/bmj.n71, PMID: 33782057 PMC8005924

[24] Higgins JP Altman DG Gøtzsche PC Jüni P Moher D Oxman AD . The cochrane collaboration’s tool for assessing risk of bias in randomised trials. BMJ. (2011) 343:d5928. doi: 10.1136/bmj.d5928, PMID: 22008217 PMC3196245

[25] Sterne J Savović J Page MJ Elbers RG Blencowe NS Boutron I . Rob 2: a revised tool for assessing risk of bias in randomised trials. BMJ. (2019) 366:l4898. doi: 10.1136/bmj.l4898 31462531

[26] Jackson D Riley R White IR. Multivariate meta-analysis: potential and promise. Stat Med. (2011) 30:2481–98. doi: 10.1002/sim.4172, PMID: 21268052 PMC3470931

[27] Salanti G Ades AE Ioannidis JP. Graphical methods and numerical summaries for presenting results from multiple-treatment meta-analysis: an overview and tutorial. J Clin Epidemiol. (2011) 64:163–71. doi: 10.1016/j.jclinepi.2010.03.016, PMID: 20688472

[28] Chaimani A Higgins JP Mavridis D Spyridonos P Salanti G. Graphical tools for network meta-analysis in stata. PLoS One. (2013) 8:e76654. doi: 10.1371/journal.pone.0076654, PMID: 24098547 PMC3789683

[29] Puhan MA Schünemann HJ Murad MH Li T Brignardello-Petersen R Singh JA . A grade working group approach for rating the quality of treatment effect estimates from network meta-analysis. BMJ. (2014) 349:g5630. doi: 10.1136/bmj.g5630 25252733

[30] Hsiung WT Chang YC Yeh ML Chang YH. Acupressure improves the postoperative comfort of gastric cancer patients: a randomised controlled trial. Complement Ther Med. (2015) 23:339–46. doi: 10.1016/j.ctim.2015.03.010 26051568

[31] Ding YQ Huang T. Electroacupuncture to promote rapid recovery of patients after distal radical surgery for gastric cancer: a randomised controlled double-blind study. J Baotou Med Coll. (2021) 37:107–10. doi: 10.16833/j.cnki.jbmc.2021.03.029

[32] Huang WJ Duan PB Wang XQ Zhu JH Wu YH Sun L. A study on the effect of auricular acupressure on the recovery of gastrointestinal function in postoperative gastric cancer patients. J Nurs Manag. (2014) 14:827–9.

[33] Li L Xu G. Effects of acupoint injection combined with auricular acupressure on gastrointestinal function and immune function of gastric cancer patients after surgery. Chinese Contemp Med. (2016) 23:128–31.

[34] Liang HH Bian XM Wang CL Shen XD Su Q Chang YR. Clinical study on the treatment of postoperative gastrointestinal dysfunction after gastric cancer surgery by balancing fire cupping combined with moxibustion in spleen and stomach cold type. New Chinese Med. (2020) 52:156–8. doi: 10.13457/j.cnki.jncm.2020.01.045

[35] Lin XM Quan XM Lin YR Fu MH. The effect of Wu Zhu medicinal ironing combined with electroacupuncture on the recovery of gastrointestinal function after radical surgery for gastric cancer. J Nurse Adv. (2015) 30:1963–5.

[36] Qian CL Liu H Zhang J Qiu WJ Shen ZY Sun JH. Clinical study of acupuncture at the lower meridian point to promote functional recovery after gastric cancer surgery. Shanghai J Acupunc Moxibust. (2017) 36:1044–8. doi: 10.13460/j.issn.1005-0957.2017.09.1044

[37] Song CY Li Z Yang YJ Jin H Shi GH. Clinical observation on acupoint massage to promote the recovery of gastrointestinal function in patients after radical gastric cancer surgery. Shanghai J Tradit Chin Med. (2019):57–9.

[38] Tan P You JH Chen Q Cai FY Zhou JX Huang XF. Effects of auricular acupoint pressure beans on gastrointestinal function in elderly postoperative patients with gastric cancer. Nurs Res. (2017):4562–4.

[39] Wu J Zhang H Mao F. Effects of warm acupuncture combined with acupoint massage on serum gastrin, gastric motility and gastrointestinal function in gastric cancer patients after surgery. China Clin Res. (2021) 34:232–5. doi: 10.13429/j.cnki.cjcr.2021.02.022

[40] Xu R Xiu MN Li M Fu HX Zou JR. A clinical study of box moxibustion and foot bath with Qi and viscera regulating formula to promote the recovery of gastrointestinal function in postoperative gastric cancer patients. Jiangsu Tradit Chinese Med. (2017) 49:62–4.

[41] Ye GD Pan AX Xu HT. Study on the value of auricular acupoint pressure in promoting rapid recovery of gastrointestinal function in gastric cancer patients after surgery. China Modern Physician. (2018) 56:153–6.

[42] Zhang FZ Yu CJ Huang K Xu YJ. Effects of acupressure combined with auricular pressure pills on the recovery of gastrointestinal function after gastric cancer surgery. Sichuan Tradit Chinese Med. (2022) 40:214–7.

[43] Chen Y. Effect of acupuncture and moxibustion combined with functional exercise programme on gastrointestinal function in patients after radical gastric cancer surgery. Henan Tradit Chinese Med. (2017) 37:1477–9.

[44] Mei XL. Clinical observation on 50 cases of gastric Cancer treated with combination of traditional Chinese and Western medicine in early postoperative period. J Qiqihar Med Coll. (2013) 34:2386–7.

[45] Ren D. Clinical efficacy of moxibustion in treating postoperative abdominal distension after gastric cancer. New Chinese Med. (2016) 48:184–5. doi: 10.13457/j.cnki.jncm.2016.11.080

[46] Sun L Duan PB Huang WJ Mei SJ Wang XQ Yang LH . Auricular acupressure to promote the recovery of gastrointestinal function after gastric cancer surgery. Chinese J Integr Med Digest. (2014) 22:239–41.

[47] Xie XP Xu ZY Jin D. Effects of auricular acupressure with Wang Bu Liuhang seeds on the recovery of gastrointestinal function and serum inflammatory indexes in postoperative gastric cancer patients. J Youjiang Med Coll National. (2020) 42:621–3.

[48] Yang L Wu XC Huang JB. Effect of qi regulating and internal organs acupuncture on postoperative gastrointestinal function recovery in patients undergoing radical gastric cancer surgery. New Chinese Med. (2022) 54:143–7. doi: 10.13457/j.cnki.jncm.2022.09.033

[49] Yuan WY Yang WN Zhu FH. Acupuncture and moxibustion combined with Chinese herbal patch at Shenque point to promote the recovery of gastrointestinal function in gastric cancer patients after surgery. J Shanxi Univ Tradit Chinese Med. (2021) 44:93–6. doi: 10.13424/j.cnki.jsctcm.2021.06.019

[50] Yuan YH Shen XF Zhao YP. Effects of auricular acupressure combined with acupressure on postoperative pain and gastrointestinal function in gastric cancer. Integr Nurs Chinese Western Med. (2018) 4:70–3.

[51] Nan N Zhang Y Lu HS. Regulation of gastrointestinal function and serum gastrointestinal hormones by auricular acupressure after radical surgery for gastric cancer. Modern Physicians China. (2018) 56:92–4.

[52] Wu XL Miao D Zhang C Liu J Gong GW Wang G . Evaluation of the clinical effect of Sheng’s evening needle row stabbing method of acupuncture on the upper Giant void to intervene in the recovery of gastrointestinal function after gastric cancer surgery. Chinese J Tradit Chinese Med. (2020) 35:5291–4.

[53] Mbuagbaw L Rochwerg B Jaeschke R Heels-Andsell D Alhazzani W Thabane L . Approaches to interpreting and choosing the best treatments in network meta-analyses. Syst Rev. (2017) 6:79. doi: 10.1186/s13643-017-0473-z, PMID: 28403893 PMC5389085

[54] Lin D Ou Y Li L Wu K Zhang Q Yan J . Acupuncture for postoperative gastrointestinal dysfunction in cancer: a systematic review and meta-analysis. Front Oncol. (2023) 13:1184228. doi: 10.3389/fonc.2023.1184228, PMID: 37361600 PMC10289226

[55] Wang Y Wang L Ni X Jiang M Zhao L. Effect of acupuncture therapy for postoperative gastrointestinal dysfunction in gastric and colorectal cancers: an umbrella review. Front Oncol. (2024) 14:1291524. doi: 10.3389/fonc.2024.1291524, PMID: 38375156 PMC10876295

[56] Wang X Shi H Shang H He W Chen S Litscher G . Effect of electroacupuncture at st36 on gastric-related neurons in spinal dorsal horn and nucleus tractus solitarius. Evid Based Complement Alternat Med. (2013) 2013:912898. doi: 10.1155/2013/912898, PMID: 24191172 PMC3804039

[57] Xia X Zhang Z Zhu C Ni B Wang S Yang S . Neutrophil extracellular traps promote metastasis in gastric cancer patients with postoperative abdominal infectious complications. Nat Commun. (2022) 13:1017. doi: 10.1038/s41467-022-28492-5, PMID: 35197446 PMC8866499

[58] Snigdha S Ha K Tsai P Dinan TG Bartos JD Shahid M. Probiotics: potential novel therapeutics for microbiota-gut-brain axis dysfunction across gender and lifespan. Pharmacol Ther. (2022) 231:107978. doi: 10.1016/j.pharmthera.2021.107978, PMID: 34492236

[59] Lan L Zeng F Liu GJ Ying L Wu X Liu M . Acupuncture for functional dyspepsia. Cochrane Database Syst Rev. (2014) 2014:CD008487. doi: 10.1002/14651858.CD008487.pub2, PMID: 25306866 PMC10558101

[60] Vukelic M Qing X Redecha P Koo G Salmon JE. Cholinergic receptors modulate immune complex-induced inflammation *in vitro* and *in vivo*. J Immunol. (2013) 191:1800–7. doi: 10.4049/jimmunol.1203467, PMID: 23851693

[61] Chen X Wang Y Tong L Wu LB Li N Chu HR. Effects of moxibustion on the expression of substance p and vasoactive intestinal peptide in colon and hypothalamic tissues of rats with diarrhea-type irritable bowel syndrome model. J Gansu Univ Tradit Chinese Med. (2021) 38:1–5. doi: 10.16841/j.issn1003-8450.2021.01.01

[62] Xiang J Chen G Ouyang LZ Li F Xiang LT Chen Y . Effects of moxibustion on the content of endogenous protective factors and expression of related proteins in rats with gastric mucosal injury. J Beijing Univ Chinese Med. (2016) 39:406–12.

[63] Sha T Gao LL Zhang CH Zheng JG Meng ZH. An update on acupuncture point injection. QJM. (2016) 109:639–41. doi: 10.1093/qjmed/hcw055 27083985

[64] Waldum HL Sagatun L Mjønes P. Gastrin and gastric cancer. Front Endocrinol. (2017) 8:1. doi: 10.3389/fendo.2017.00001 PMC5239792 28144230

[65] Shi JF Li HL Wang HL Wu HY. Literature evaluation of Astragalus and its active ingredients against digestive tract tumours. Western Tradit Chinese Med. (2014) 27:44–6.

[66] You X Wang Y Wu J Liu Q Liu Y Qian Y . Zusanli (st36) acupoint injection with neostigmine for paralytic postoperative ileus following radical gastrectomy for gastric cancer: a randomized clinical trial. J Cancer. (2018) 9:2266–74. doi: 10.7150/jca.24767, PMID: 30026821 PMC6036725

[67] Tseng YL Hsu CH Tseng HC. Using acupressure to improve abdominal bloating in a hemicolectomy patient: a nursing experience. Hu Li Za Zhi. (2015) 62:96–102. doi: 10.6224/JN62.5.96, PMID: 26507632

[68] Liu Y Tang W Gong S Chan C. A systematic review and meta-analysis of acupressure for postoperative gastrointestinal symptoms among abdominal surgery patients. Am J Chin Med. (2017) 45:1127–45. doi: 10.1142/S0192415X17500616, PMID: 28830215

[69] Gao SP. Clinical study of acupoint massage on abdominal distension and constipation after gynecologic surgery. China Maternal Child Health. (2013) 28:2480–2.

[70] Lin XL Chen JW Zhao JY Tan C Chen RY. Clinical study of acupressure to promote the recovery of gastrointestinal function after cardiac surgery. World J Integr Med. (2016) 11:682–4. doi: 10.13935/j.cnki.sjzx.160521

[71] Jin HY Zhai D Jin SE Zhou R Zhang JX Fang Q. Effects of acupressure combined with external application of traditional Chinese medicine foot-sanli on gastric actin and gastrin in patients undergoing colorectal surgery. Chinese Family Med. (2019) 17:1014–7. doi: 10.16766/j.cnki.issn.1674-4152.000850

[72] Ao XR Ma KM Liao C Yu J Shen GX. Effects of electroacupuncture therapy on gastrointestinal function and serum gastrin level in abdominal surgery patients. Shaanxi Tradit Chinese Med. (2017) 38:1130–1.

[73] Wu JF Wei X Qiu HS. Experimental study of electroacupuncture for the treatment of postoperative intestinal paralysis in rats after abdominal surgery. Zhejiang J Integr Chinese Western Med. (2014) 24:675–7.

[74] Santucci NR Velez A. Physiology of lower gastrointestinal tract. Aliment Pharmacol Ther. (2024) 60:S1–S19. doi: 10.1111/apt.17900 38924125 PMC12278992

[75] Al-Bedah A Elsubai IS Qureshi NA Aboushanab TS Ali G El-Olemy AT . The medical perspective of cupping therapy: effects and mechanisms of action. J Tradit Complement Med. (2019) 9:90–7. doi: 10.1016/j.jtcme.2018.03.003, PMID: 30963043 PMC6435947

[76] Ying X Yu HX Zhang Y Shen Q Sun QH. Clinical observation of moxibustion combined with cupping to promote the recovery of intestinal peristalsis after general anesthesia abdominal surgery. Chinese J Tradit Chinese Med. (2013) 28:3623–5.

[77] Yin C Fang Y Yao D Zhang X. Influencing mechanism of cupping moxibustion on gastrointestinal function and immune function in patients with functional diarrhea. Cell Mol Biol. (2022) 68:98–104. doi: 10.14715/cmb/2022.68.6.16 36227672

[78] Yang JW Shao JK Wang Y Liu Q Liang JW Yan SY . Effect of acupuncture on postoperative ileus after laparoscopic elective colorectal surgery: a prospective, randomised, controlled trial. Eclinicalmedicine. (2022) 49:101472. doi: 10.1016/j.eclinm.2022.101472, PMID: 35747183 PMC9156985

[79] Noguchi E. Acupuncture regulates gut motility and secretion via nerve reflexes. Auton Neurosci. (2010) 156:15–8. doi: 10.1016/j.autneu.2010.06.010 20663717

[80] Liang C Wang KY Gong MR Li Q Yu Z Xu B. Electro-acupuncture at st37 and st25 induce different effects on colonic motility via the enteric nervous system by affecting excitatory and inhibitory neurons. Neurogastroenterol Motil. (2018) 30:e13318. doi: 10.1111/nmo.13318, PMID: 29488287

[81] Huang YX Wang JJ Wang XB Wang J. Neural action mechanism of electroacupuncture at gastric meridian points in regulating gastric motility. J Gastroenterol Hepatol. (2004):358–62.

[82] Li H Wang YP. Effect of auricular acupuncture on gastrointestinal motility and its relationship with vagal activity. Acupunct Med. (2013) 31:57–64. doi: 10.1136/acupmed-2012-010173, PMID: 23211189

[83] Lee CH Kim DK Yook TH Sasaki M Kitamura N. Effectiveness of electroacupuncture at zusanli (st36) on the immunohistochemical density of enteroendocrine cells related to gastrointestinal function. J Acupunct Meridian Stud. (2012) 5:63–71. doi: 10.1016/j.jams.2012.01.002, PMID: 22483184

[84] Lu MJ Yu Z He Y Yin Y Xu B. Electroacupuncture at st36 modulates gastric motility via vagovagal and sympathetic reflexes in rats. World J Gastroenterol. (2019) 25:2315–26. doi: 10.3748/wjg.v25.i19.2315, PMID: 31148903 PMC6529886

[85] Murakami H Li S Foreman R Yin J Hirai T Chen J. Ameliorating effects of electroacupuncture on dysmotility, inflammation, and pain mediated via the autonomic mechanism in a rat model of postoperative ileus. J Neurogastroenterol Motil. (2019) 25:286–99. doi: 10.5056/jnm18094, PMID: 30827069 PMC6474706

[86] Shi L Fang J Zhao J Liu G Zhao Q Zhang J . Comparison of the therapeutic effects of acupuncture at pc6 and st36 for chronic myocardial ischemia. Evid Based Complement Alternat Med. (2017) 2017:7358059. doi: 10.1155/2017/7358059, PMID: 28900462 PMC5576407

[87] Li WJ Gao C An LX Ji YW Xue FS Du Y. Perioperative transcutaneous electrical acupoint stimulation for improving postoperative gastrointestinal function: a randomized controlled trial. J Integr Med. (2021) 19:211–8. doi: 10.1016/j.joim.2021.01.005, PMID: 33495134

[88] Chen SY Wei MQ. Effect of acupuncture on the recovery of gastrointestinal function after gastric cancer surgery - a systematic evaluation and meta-analysis of randomized controlled trials. China Tradit Chinese Med Modern Dist Educ. (2022) 20:59–61.

