# Supplementary material for: Acupuncture and moxibustion as adjunctive therapy for postoperative gastrointestinal dysfunction in gastric cancer: a systematic review and network meta-analysis
Source: Front Med (Lausanne). 2024 Dec 11;11:1464749. doi: 10.3389/fmed.2024.1464749 (PMC11668611; doi:10.3389/fmed.2024.1464749)
Supplement: Supplementary file 1 [file Data_Sheet_1.docx]

Supplementary material

**Supplementary** [**Table S1| Search Strategy for the PubMed Database**](#_Toc119062184) **1**

**Supplementary** [**Table S2| Search Strategy for the Web of Science Database**](#_Toc119062184) **2**

**Supplementary** [**Table S3| Search Strategy for the Embase Database**](#_Toc119062184) **3**

**Supplementary** [**Table S4| Search Strategy for the Cochrane Library Database**](#_Toc119062184) **4**

**Supplementary** [**Table S5| Search Strategy for the CNKI Database**](#_Toc119062184) **5**

**Supplementary** [**Table S6| Search Strategy for the VIP Database**](#_Toc119062184) **5**

**Supplementary** [**Table S7| Search Strategy for the Wan Fan Database**](#_Toc119062184) **5**

**Supplementary** [**Table S8| Search Strategy for the CBM Database**](#_Toc119062184) **5**

**Supplementary** [**Table S9| Head-to-head comparisons for an TFBS of different interventions**](#_Toc119062187) **6**

**Supplementary** [**Table S10| Head-to-head comparisons for an TFD of different interventions**](#_Toc119062187) **8**

**Supplementary** [**Table S11| Head-to-head comparisons for an TFF of different interventions 1**](#_Toc119062188)**0**

**Supplementary** [**Table S12| Evidence Quality of TFBS**](#_Toc119062188) **12**

**Supplementary** [**Table S13| Evidence Quality of TFD**](#_Toc119062188) **15**

**Supplementary** [**Table S14| Evidence Quality of TFF**](#_Toc119062189) **19**

**Supplementary** [**S15| STATA 15.1 graphic code and official website link in this study**](#_Toc119062189) **20**

Supplementary Table S1. Search Strategy for the PubMed Database

| #1 | **“Acupuncture Therapy” [MeSH Terms]** |
| --- | --- |
| #2 | **(((((((((Acupuncture Treatment [Title/Abstract]) OR (Acupuncture Treatments [Title/Abstract])) OR (Treatment, Acupuncture [Title/Abstract])) OR (Therapy, Acupuncture [Title/Abstract])) OR (Pharmacoacupuncture Treatment [Title/Abstract])) OR (Treatment, Pharmacoacupuncture[Title/Abstract])) OR (Pharmacoacupuncture Therapy[Title/Abstract])) OR (Therapy, Pharmacoacupuncture[Title/Abstract])) OR (Acupotomy[Title/Abstract])) OR (Acupotomies[Title/Abstract])** |
| #3 | **(acupunctur*[Title/Abstract] OR electroacupunctur*[Title/Abstract] OR electro-acupunctur*[Title/Abstract] OR acupoint*[Title/Abstract] OR electric acupunctur*[Title/Abstract] OR acu-point*[Title/Abstract] OR acupress*[Title/Abstract])** |
| #4 | **(meridian*[Title/Abstract]) OR (non-meridian [Title/Abstract] OR trigger [Title/Abstract])** |
| #5 | **(((((((((((((zhenjiu[Title/Abstract]) OR (zhen jiu[Title/Abstract])) OR (zhenci[Title/Abstract])) OR (zhen ci[Title/Abstract])) OR (cizhen[Title/Abstract])) OR (dianzhen[Title/Abstract])) OR (dian zhen[Title/Abstract])) OR (zhen ya[Title/Abstract])) OR (er zhen[Title/Abstract])) OR (ti zhen[Title/Abstract])) OR (she zhen[Title/Abstract])) OR (tou pi zhen[Title/Abstract])) OR (zue wei[Title/Abstract])) OR (pi nei zhen[Title/Abstract])** |
| #6 | **((((((((((((((moxibustion[Title/Abstract]) OR (fire needle[Title/Abstract])) OR (fire acupuncture[Title/Abstract])) OR (warm acupuncture[Title/Abstract])) OR (warm needle[Title/Abstract])) OR (needle warming moxibustion[Title/Abstract])) OR (heat sensitive moxibustion[Title/Abstract])) OR (ear needle[Title/Abstract])) OR (auricular needle[Title/Abstract])) OR (wrist ankle needle[Title/Abstract])) OR (acupoint embedding[Title/Abstract])) OR (acupoint injection[Title/Abstract])) OR (acupoint sticking[Title/Abstract])) OR (intradermal needling[Title/Abstract])) OR (scalp acupuncture[Title/Abstract])** |
| #7 | **(((((((((((((transcutaneous electric$ nerve stimulation[Title/Abstract]) OR (percutaneous electric$ stimulation[Title/Abstract])) OR (transcutaneous electric$ stimulation[Title/Abstract])) OR (transdermal electrostimulation[Title/Abstract])) OR (transcutaneous electric$ acupoint stimulation[Title/Abstract])) OR (neuromuscular electric$ stimulation[Title/Abstract])) OR (functional electric$ stimulation[Title/Abstract])) OR (transcranial direct current stimulation[Title/Abstract])) OR (TENS[Title/Abstract])) OR (PENS[Title/Abstract])) OR (TEAS[Title/Abstract])) OR (NMES[Title/Abstract])) OR (FES[Title/Abstract])) OR (tDCS[Title/Abstract])** |
| #8 | **#1 OR #2 OR #3 OR #4 OR #5 OR #6 OR #7** |
| #9 | **“Stomach Neoplasms” [MeSH Terms]** |
| #10 | **((((((((((((Neoplasm, Stomach[Title/Abstract])) OR (Stomach Neoplasm[Title/Abstract])) OR (Gastric Neoplasms[Title/Abstract])) OR (Neoplasm, Gastric[Title/Abstract])) OR (Cancer of Stomach[Title/Abstract])) OR (Stomach Cancers[Title/Abstract])) OR (Gastric Cancer[Title/Abstract])) OR (Cancer, Gastric[Title/Abstract])) OR (Stomach Cancer[Title/Abstract])) OR (Cancer, Stomach[Title/Abstract])) OR (Cancer of the Stomach[Title/Abstract])) OR (Gastric Cancer, Familial Diffuse[Title/Abstract])** |
| #11 | **#9 or #10** |
| #12 | **“operation [MeSH Terms]”** |
| #13 | **(((((operation [MeSH Terms])) OR (Surgery [Title/Abstract])) OR (postoperation[Title/Abstract])) OR (Postoperative[Title/Abstract])) OR (Post operation[Title/Abstract])** |
| #14 | **#12 or #13** |
| #15 | **((((((((((((((groups[Title/Abstract]) OR (trial[Title/Abstract])) OR (randomized controlled trial[Title/Abstract])) OR (controlled clinical trial[Title/Abstract])) OR (random allocation[Title/Abstract])) OR (randomized[Title/Abstract])) OR (randomly[Title/Abstract])) OR (double-blind method[Title/Abstract])) OR (single-blind method[Title/Abstract])) OR (clinical trial[Title/Abstract])) OR ((double$ adj blind$[Title/Abstract])) OR (placebo[Title/Abstract])) OR (randomized[Title/Abstract])) OR (Randomized Controlled Trial[Publication Type])) OR (controlled clinical trial[Publication Type])** |
| #16 | **#8 and #11 and #14 and #15** |

Supplementary Table S2. Search strategy for Web of Science Database

| #1 | **TS= (Acupuncture Therapy)** |
| --- | --- |
| #2 | **AB=(Acupuncture Treatment OR Acupuncture Treatments OR Treatment, Acupuncture OR Therapy, Acupuncture OR Pharmacoacupuncture Treatment OR Treatment, Pharmacoacupuncture OR Pharmacoacupuncture Therapy OR Therapy, Pharmacoacupuncture OR Acupotomy OR Acupotomies OR acupunctur* OR electroacupunctur* OR electro-acupunctur* OR acupoint* OR electric acupunctur* OR acu-point* OR acupress* OR meridian* OR non-meridian OR trigger OR zhenjiu OR zhen jiu OR zhenci OR zhen ci OR cizhen OR dianzhen OR dian zhen OR zhen ya OR er zhen OR ti zhen OR she zhen OR tou pi zhen OR zue wei OR pi nei zhen OR ching lo OR jing luo OR jinglo OR moxibustion OR fire needle OR fire acupuncture OR warm acupuncture OR warm needle OR needle warming moxibustion OR heat sensitive moxibustion OR ear needle OR auricular needle OR wrist ankle needle OR acupoint embedding OR acupoint injection OR acupoint sticking OR intradermal needling OR scalp acupuncture OR transcutaneous electric$ nerve stimulation OR percutaneous electric$ stimulation OR transcutaneous electric$ stimulation OR transdermal electrostimulation OR transcutaneous electric$ acupoint stimulation OR neuromuscular electric$ stimulation OR functional electric$ stimulation OR transcranial direct current stimulation OR TENS OR PENS OR TEAS OR NMES OR FES OR tDCS)** |
| #3 | **#1 or #2** |
| #4 | **TS=(Stomach Neoplasms)** |
| #5 | **AB= (Neoplasm, Stomach** **OR Stomach Neoplasm** **OR Gastric Neoplasms OR** **Neoplasm, Gastric** **OR Cancer of Stomach** **OR Stomach Cancers** **OR Gastric Cancer** **OR Cancer, Gastric** **OR** **Stomach Cancer OR Cancer, Stomach** **OR Cancer of the Stomach OR** **Gastric Cancer, Familial Diffuse)** |
| #6 | **#4 or #5** |
| #7 | **TS= (operation)** |
| #8 | **AB = (operation** **OR Surgery** **OR postoperation** **OR** **Postoperative** **OR** **Post operation** **)** |
| #9 | **#7 or #8** |
| #10 | **AB=(groups OR trial OR "randomized controlled trial" OR "controlled clinical trial" OR "random allocation" OR randomized OR "randomly" OR "double-blind method" OR "single-blind method" OR "clinical trial" OR “double$ adj blind$” OR placebo OR randomized OR "Randomized Controlled Trial" OR “controlled clinical trial”)** |
| #11 | **#3 and #6 and #9 and #10** |

Supplementary Table S3. Search strategy for Embase Database

| #1 | exp acupuncture therapy/ |
| --- | --- |
| #2 | acupunctur$ or electroacupunctur$ or electro-acupunctur$ or electric acupunctur$ or acupoint$ or acu-point$ or acupress$).mp. |
| #3 | ((meridian$ or non-meridian or trigger) adj10 point$).tw. |
| #4 | (zhenjiu or zhen jiu or zhenci or zhen ci or cizhen or dianzhen or dian zhen or zhen ya or er zhen or ti zhen or she zhen or tou pi zhen or zue wei).tw. |
| #5 | ((ching adj2 lo) or (jing adj2 luo) or jinglo).tw. |
| #6 | (moxibustion or fire needle or fire acupuncture or warm acupuncture or warm needle or needle warming moxibustion or heat sensitive moxibustion or ear needle or auricular needle or wrist ankle needle or acupoint embedding or acupoint injection or acupoint sticking).tw. |
| #7 | (transcutaneous electric$ nerve stimulation or percutaneous electric$ stimulation or transcutaneous electric$ stimulation or transdermal electrostimulation or transcutaneous electric$ acupoint stimulation or neuromuscular electric$ stimulation or functional electric$ stimulation or transcranial direct current stimulation or TENS or PENS or TEAS or NMES or FES or tDCS). mp. |
| #8 | #1 or #2 or #3 or #4 or #5 or #6 or #7 |
| #9 | exp **Stomach Neoplasms**/ |
| #10 | (**Neoplasm, Stomach** **OR** **Stomach Neoplasm** **OR Gastric Neoplasms OR Neoplasm, Gastric** **OR Cancer of Stomach** **OR Stomach Cancers** **OR Gastric Cancer** **OR Cancer, Gastric** **OR** **Stomach Cancer OR Cancer, Stomach** **OR Cancer of the Stomach OR** **Gastric Cancer, Familial Diffuse**) tw. |
| #11 | #9 or #10 |
| #12 | exp **operation** / |
| #13 | (**operation** **OR Surgery** **OR postoperation** **OR** **Postoperative** **OR** **Post operation**) tw. |
| #14 | #12 or #13 |
| #15 | (groups OR trial OR randomized controlled trial OR controlled clinical trial OR random allocation OR randomized OR randomly OR double-blind method OR single-blind method OR clinical trial OR double$ adj blind$ OR placebo OR randomized OR Randomized Controlled Trial OR controlled clinical trial) tw. |
| #16 | #8 or #11 or #14 |

Supplementary Table S4. Search strategy for the Cochrane Library Database

| #1 | MeSH descriptor: [Acupuncture Therapy] explode all trees |
| --- | --- |
| #2 | (Acupuncture Treatment OR Acupuncture Treatments OR Treatment, Acupuncture OR Therapy, Acupuncture OR Pharmacoacupuncture Treatment OR Treatment, Pharmacoacupuncture OR Pharmacoacupuncture Therapy OR Therapy, Pharmacoacupuncture OR Acupotomy OR Acupotomies OR acupunctur* OR electroacupunctur* OR electro-acupunctur* OR acupoint* OR electric acupunctur* OR acu-point* OR acupress* OR meridian* OR non-meridian OR trigger OR zhenjiu OR zhen jiu OR zhenci OR zhen ci OR cizhen OR dianzhen OR dian zhen OR zhen ya OR er zhen OR ti zhen OR she zhen OR tou pi zhen OR zue wei OR pi nei zhen OR ching lo OR jing luo OR jinglo OR moxibustion OR fire needle OR fire acupuncture OR warm acupuncture OR warm needle OR needle warming moxibustion OR heat sensitive moxibustion OR ear needle OR auricular needle OR wrist ankle needle OR acupoint embedding OR acupoint injection OR acupoint sticking OR intradermal needling OR scalp acupuncture OR transcutaneous electric$ nerve stimulation OR percutaneous electric$ stimulation OR transcutaneous electric$ stimulation OR transdermal electrostimulation OR transcutaneous electric$ acupoint stimulation OR neuromuscular electric$ stimulation OR functional electric$ stimulatio |
| #3 | #1 or #2 |
| #4 | MeSH descriptor: [**Stomach Neoplasms**] explode all trees |
| #5 | (**Neoplasm, Stomach** **OR Stomach Neoplasm** **OR Gastric Neoplasms OR Neoplasm, Gastric** **OR Cancer of Stomach** **OR Stomach Cancers** **OR Gastric Cancer** **OR Cancer, Gastric** **OR** **Stomach Cancer OR Cancer, Stomach** **OR Cancer of the Stomach OR** **Gastric Cancer, Familial Diffuse**):ti,ab in Trials |
| #6 | #4 or #5 |
| #7 | MeSH descriptor: [**operation**] explode all trees |
| #8 | (**operation** **OR Surgery** **OR postoperation** **OR** **Postoperative** **OR** **Post operation**):ti,ab in Trials |
| #9 | #7 or #8 |
| #10 | (groups OR trial OR "randomized controlled trial" OR "controlled clinical trial" OR "random allocation" OR randomized OR "randomly" OR "double-blind method" OR "single-blind method" OR "clinical trial" OR “double$ adj blind$” OR placebo OR randomized OR "Randomized Controlled Trial" OR “controlled clinical trial”) :ti,ab in Trials |
| #11 | #3 and #6 and #9 and #10 |

**Supplementary Table S5. Search strategy for CNKI Database**

| #1 | 主题（SU）=针灸 + 针刺 + 电针 + 艾灸 + 灸法 + 温针灸 + 火针 + 耳针 + 耳穴 + 眼针 + 皮肤针 + 头针 + 皮内针 + 穴位敷贴 + 穴位注射 + 穴位埋线 + 腕踝针 + 电刺激疗法 + 经皮电刺激 + 经皮神经电刺激 + 经颅直流电刺激 + 经颅电刺激 + 经皮穴位电刺激 + TENS + PENS |
| --- | --- |
| #2 | 主题（SU）=胃部肿瘤 + 胃癌 |
| #3 | 主题（SU）=手术 + 术后 |
| #4 | 主题（SU）=随机对照试验 + RCT + 随机 + 对照 + 临床研究 + 临床观察 |
| #5 | #1 AND #2 AND #3AND #4 |

**Supplementary Table S6. Search strategy for VIP Database**

| #1 | 题名或关键词：:针灸 + 针刺 + 电针 + 艾灸 + 灸法 + 温针灸 + 火针 + 耳针 + 耳穴 + 眼针 + 皮肤针 + 头针 + 皮内针 + 穴位敷贴 + 穴位注射 + 穴位埋线 + 腕踝针 + 电刺激疗法 + 经皮电刺激 + 经皮神经电刺激 + 经颅直流电刺激 + 经颅电刺激 + 经皮穴位电刺激 + TENS + PENS |
| --- | --- |
| #2 | 摘要：胃部肿瘤 + 胃癌 |
| #3 | 摘要：手术 + 术后 |
| #4 | 摘要：随机对照试验 + RCT + 随机 + 对照 + 临床研究 + 临床观察 |
| #5 | #1 AND #2 AND #3AND #4 |

**Supplementary Table S7. Search strategy for Wan Fang** **Database**

| #1 | 主题：针灸 + 针刺 + 电针 + 艾灸 + 灸法 + 温针灸 + 火针 + 耳针 + 耳穴 + 眼针 + 皮肤针 + 头针 + 皮内针 + 穴位敷贴 + 穴位注射 + 穴位埋线 + 腕踝针 + 电刺激疗法 + 经皮电刺激 + 经皮神经电刺激 + 经颅直流电刺激 + 经颅电刺激 + 经皮穴位电刺激 + TENS + PENS |
| --- | --- |
| #2 | 主题：胃部肿瘤 + 胃癌 |
| #3 | 主题：手术 + 术后 |
| #4 | 主题：随机对照试验 or RCT or 随机 or 对照 or 临床研究 or 临床观察 |
| #5 | #1 AND #2 AND #3AND #4 |

**Supplementary Table S8. Search strategy for CBM** **Database**

| #1 | 常用字段：针灸 OR 针刺 OR 电针 OR 艾灸 OR 灸法 OR 温针灸 OR 火针 OR 耳针 OR 耳穴 OR 眼针 OR 皮肤针 OR 头针 OR 皮内针 OR 穴位敷贴 OR 穴位注射 OR 穴位埋线 OR 腕踝针 OR 电刺激疗法 OR 经皮电刺激 OR 经皮神经电刺激 OR 经颅直流电刺激 OR 经颅电刺激 OR 经皮穴位电刺激 OR TENS OR PENS |
| --- | --- |
| #2 | 常用字段：胃部肿瘤 + 胃癌 |
| #3 | 常用字段：手术 + 术后 |
| #4 | 常用字段：随机对照试验 OR RCT OR 随机 OR 对照 OR 临床研究 OR 临床观察 |
| #5 | #1 AND #2 AND #3AND #4 |

Supplementary Table S9. Head-to-head comparisons for TFBS of the different interventions

| **RC+ACU** | **RC+AI+MOX** | **RC+ACUP** | **RC+AA+AI** | **RC+AI** | **RC+WA+ACUP** | **RC+PAT+ACU** | **RC+MOX+CUP** | **RC+TEAS** | **RC+AA** | **RC+MOX** | **RC+AA+ACUP** | **RC+EA+PAT** | **RC** |
| --- | --- | --- | --- | --- | --- | --- | --- | --- | --- | --- | --- | --- | --- |
| **RC+ACU** | 4.20 (-11.89,20.29) | 4.70 (-11.22,20.62) | 5.20 (-10.84,21.24) | 6.70 (-9.67,23.07) | 10.53 (-5.40,26.46) | 11.00 (-4.78,26.78) | 15.21 (-1.70,32.12) | 15.60 (-0.24,31.44) | 16.51 (4.09,28.92) | 19.66 (6.89,32.43) | 20.57 (4.77,36.37) | 23.11 (7.37,38.85) | 23.90 (12.41,35.39) |
| -4.20 (-20.29,11.89) | **RC+MOX +AI** | 0.50 (-15.26,16.26) | 1.00 (-14.89,16.89) | 2.50 (-13.71,18.71) | 6.33 (-9.44,22.10) | 6.80 (-8.82,22.42) | 11.01 (-5.76,27.77) | 11.40 (-4.28,27.08) | 12.31 (0.10,24.52) | 15.46 (2.89,28.03) | 16.37 (0.73,32.01) | 18.91 (3.33,34.49) | 19.70 (8.43,30.97) |
| -4.70 (-20.62,11.22) | -0.50 (-16.26,15.26) | **RC+ACUP** | 0.50 (-15.22,16.22) | 2.00 (-14.04,18.04) | 5.83 (-9.77,21.43) | 6.30 (-9.14,21.74) | 10.51 (-6.09,27.11) | 10.90 (-4.61,26.41) | 11.81 (-0.18,23.80) | 14.96 (2.60,27.31) | 15.87 (0.40,31.34) | 18.41 (3.00,33.82) | 19.20 (8.18,30.22) |
| -5.20 (-21.24,10.84) | -1.00 (-16.89,14.89) | -0.50 (-16.22,15.22) | **RC+AA +AI** | 1.50 (-14.67,17.67) | 5.33 (-10.39,21.05) | 5.80 (-9.77,21.37) | 10.01 (-6.71,26.73) | 10.40 (-5.24,26.04) | 11.31 (-0.84,23.46) | 14.46 (1.94,26.97) | 15.37 (-0.23,30.97) | 17.91 (2.37,33.45) | 18.70 (7.50,29.90) |
| -6.70 (-23.07,9.67) | -2.50 (-18.71,13.71) | -2.00 (-18.04,14.04) | -1.50 (-17.67,14.67) | **RC+AI** | 3.83 (-12.22,19.88) | 4.30 (-11.60,20.20) | 8.51 (-8.52,25.54) | 8.90 (-7.07,24.87) | 9.81 (-2.76,22.38) | 12.96 (0.03,25.88) | 13.87 (-2.06,29.80) | 16.41 (0.54,32.28) | 17.20 (5.54,28.86) |
| -10.53 (-26.46,5.40) | -6.33 (-22.10,9.44) | -5.83 (-21.43,9.77) | -5.33 (-21.05,10.39) | -3.83 (-19.88,12.22) | **RC+WA+ACUP** | 0.47 (-14.98,15.92) | 4.68 (-11.93,21.29) | 5.07 (-10.45,20.59) | 5.98 (-6.02,17.98) | 9.13 (-3.24,21.49) | 10.04 (-5.44,25.52) | 12.58 (-2.84,28.00) | 13.37 (2.34,24.40) |
| -11.00 (-26.78,4.78) | -6.80 (-22.42,8.82) | -6.30 (-21.74,9.14) | -5.80 (-21.37,9.77) | -4.30 (-20.20,11.60) | -0.47 (-15.92,14.98) | **RC+ACU+PAT** | 4.21 (-12.26,20.67) | 4.60 (-10.76,19.96) | 5.51 (-6.29,17.31) | 8.66 (-3.51,20.83) | 9.57 (-5.75,24.89) | 12.11 (-3.15,27.37) | 12.90 (2.08,23.72) |
| -15.21 (-32.12,1.70) | -11.01 (-27.77,5.76) | -10.51 (-27.11,6.09) | -10.01 (-26.73,6.71) | -8.51 (-25.54,8.52) | -4.68 (-21.29,11.93) | -4.21 (-20.67,12.26) | **RC+MOX+CUP** | 0.39 (-16.14,16.92) | 1.30 (-11.98,14.58) | 4.45 (-6.64,15.54) | 5.36 (-11.13,21.85) | 7.90 (-8.53,24.34) | 8.69 (-3.72,21.11) |
| -15.60 (-31.44,0.24) | -11.40 (-27.08,4.28) | -10.90 (-26.41,4.61) | -10.40 (-26.04,5.24) | -8.90 (-24.87,7.07) | -5.07 (-20.59,10.45) | -4.60 (-19.96,10.76) | -0.39 (-16.92,16.14) | **RC+TEAS** | 0.91 (-10.98,12.79) | 4.06 (-8.20,16.31) | 4.97 (-10.42,20.36) | 7.51 (-7.82,22.84) | 8.30 (-2.61,19.21) |
| **-16.51 (-28.92,-4.09)** | **-12.31 (-24.52,-0.10)** | -11.81 (-23.80,0.18) | -11.31 (-23.46,0.84) | -9.81 (-22.38,2.76) | -5.98 (-17.98,6.02) | -5.51 (-17.31,6.29) | -1.30 (-14.58,11.98) | -0.91 (-12.79,10.98) | **RC+AA** | 3.15 (-4.16,10.45) | 4.06 (-7.77,15.89) | 6.60 (-5.15,18.35) | 7.39 (2.68,12.10) |
| **-19.66 (-32.43,-6.89)** | **-15.46 (-28.03,-2.89)** | **-14.96 (-27.31,-2.60)** | **-14.46 (-26.97,-1.94)** | **-12.96 (-25.88,-0.03)** | -9.13 (-21.49,3.24) | -8.66 (-20.83,3.51) | -4.45 (-15.54,6.64) | -4.06 (-16.31,8.20) | -3.15 (-10.45,4.16) | **RC+MOX** | 0.91 (-11.29,13.12) | 3.45 (-8.67,15.58) | 4.24 (-1.33,9.82) |
| **-20.57 (-36.37,-4.77)** | **-16.37 (-32.01,-0.73)** | **-15.87 (-31.34,-0.40)** | -15.37 (-30.97,0.23) | -13.87 (-29.80,2.06) | -10.04 (-25.52,5.44) | -9.57 (-24.89,5.75) | -5.36 (-21.85,11.13) | -4.97 (-20.36,10.42) | -4.06 (-15.89,7.77) | -0.91 (-13.12,11.29) | **RC+AA+ACUP** | 2.54 (-12.75,17.83) | 3.33 (-7.52,14.18) |
| **-23.11 (-38.85,-7.37)** | **-18.91 (-34.49,-3.33)** | **-18.41 (-33.82,-3.00)** | **-17.91 (-33.45,-2.37)** | **-16.41 (-32.28,-0.54)** | -12.58 (-28.00,2.84) | -12.11 (-27.37,3.15) | -7.90 (-24.34,8.53) | -7.51 (-22.84,7.82) | -6.60 (-18.35,5.15) | -3.45 (-15.58,8.67) | -2.54 (-17.83,12.75) | **RC+EA+PAT** | 0.79 (-9.98,11.56) |
| **-23.90 (-35.39,-12.41)** | **-19.70 (-30.97,-8.43)** | **-19.20 (-30.22,-8.18)** | **-18.70 (-29.90,-7.50)** | **-17.20 (-28.86,-5.54)** | **-13.37 (-24.40,-2.34)** | **-12.90 (-23.72,-2.08)** | -8.69 (-21.11,3.72) | -8.30 (-19.21,2.61) | **-7.39 (-12.10,-2.68)** | -4.24 (-9.82,1.33) | -3.33 (-14.18,7.52) | -0.79 (-11.56,9.98) | **RC** |

Abbreviations: Mean difference and 95% credibility intervals between 14 interventions: indirect comparisons from network meta-analysis. Results are the MD and related 95% CIs in the row-defining treatment compared with the MD in the column-defining treatment. MD >0 favors the column-defining treatment and vice versa. The significant result is in bold and underlined. TFBS: Time of first bowel sounds; RC: routine care; RC+MOX: routine care combined with moxibustion; RC+AA: routine care combined with auricular acupuncture; RC+ACUP: routine care combined with acupressure; RC+TEAS: routine care combined with transcutaneous electrical acupoint stimulation; RC+AA+ACUP: routine care combined with auricular acupuncture and acupressure; RC+ACU: routine care combined with acupuncture; RC+MOX+CUP: routine care combined with moxibustion and cupping; RC+MOX+AI: routine care combined with moxibustion and acupoint injection; RC+WA+ACUP: routine care combined with warming acupuncture and acupressure; RC+EA+PAT: routine care combined with electro-acupuncture and point application therapy; RC+AI: routine care combined with and acupoint injection; RC+AA+AI: routine care combined with auricular acupuncture and acupoint injection; RC+ACU+PAT: routine care combined with acupuncture and point application therapy.

Supplementary Table S10. Head-to-head comparisons for an TFD of different interventions

| **RC+ACU+PAT** | **RC+MOX** | **RC+FBT** | **RC+AI** | **RC+EA+PAT** | **RC+AI+MOX** | **RC+ACU** | **RC+WA+ACUP** | **RC+AA+PAT** | **RC+TEAS** | **RC+AA+AI** | **RC+ACU+PAT** | **RC+ACUP** | **RC+ACU+FT** | **RC+PAT** | **RC+AA** | **RC+AA+ACUP** | **RC** |
| --- | --- | --- | --- | --- | --- | --- | --- | --- | --- | --- | --- | --- | --- | --- | --- | --- | --- |
| RC+MOX+CUP | 8.33 (-10.64,27.29) | 27.55 (-0.26,55.36) | 36.41 (2.26,70.57) | 34.67 (-14.79,84.14) | 40.21 (6.52,73.91) | 44.21 (13.06,75.37) | 45.53 (11.60,79.47) | 46.16 (12.72,79.61) | 47.28 (13.69,80.88) | 47.91 (14.22,81.61) | 48.54 (15.16,81.92) | 54.93 (19.60,90.27) | 55.09 (21.34,88.85) | 55.78 (22.03,89.54) | 56.60 (27.86,85.34) | 58.22 (24.83,91.62) | 61.01 (33.21,88.82) |
| -8.33 (-27.29,10.64) | RC+MOX | 19.22 (-1.12,39.56) | 28.09 (-0.32,56.50) | 26.35 (-19.34,72.03) | 31.89 (4.03,59.74) | 35.88 (11.16,60.61) | 37.21 (9.06,65.35) | 37.84 (10.28,65.39) | 38.96 (11.22,66.69) | 39.59 (11.73,67.44) | 40.22 (12.74,67.69) | 46.61 (16.79,76.43) | 46.77 (18.84,74.69) | 47.46 (19.53,75.39) | 48.27 (26.67,69.88) | 49.90 (22.40,77.39) | 52.69 (32.34,73.03) |
| -27.55 (-55.36,0.26) | -19.22 (-39.56,1.12) | RC+FBT | 8.86 (-26.07,43.80) | 7.12 (-42.88,57.13) | 12.66 (-21.82,47.15) | 16.66 (-15.35,48.67) | 17.98 (-16.74,52.70) | 18.61 (-15.63,52.85) | 19.73 (-14.65,54.12) | 20.36 (-14.12,54.85) | 20.99 (-13.19,55.17) | 27.38 (-8.71,63.47) | 27.54 (-7.00,62.09) | 28.23 (-6.31,62.78) | 29.05 (-0.62,58.72) | 30.67 (-3.52,64.86) | 33.46 (4.70,62.22) |
| -36.41 (-70.57,-2.26) | -28.09 (-56.50,0.32) | -8.86 (-43.80,26.07) | RC+AI | -1.74 (-47.20,43.72) | 3.80 (-23.68,31.28) | 7.80 (-16.51,32.10) | 9.12 (-18.66,36.90) | 9.75 (-17.43,36.93) | 10.87 (-16.49,38.23) | 11.50 (-15.98,38.98) | 12.13 (-14.97,39.23) | 18.52 (-10.95,47.99) | 18.68 (-8.88,46.24) | 19.37 (-8.19,46.93) | 20.19 (-0.94,41.31) | 21.81 (-5.31,48.93) | 24.60 (4.77,44.43) |
| -34.67 (-84.14,14.79) | -26.35 (-72.03,19.34) | -7.12 (-57.13,42.88) | 1.74 (-43.72,47.20) | RC+EA+PAT | 5.54 (-39.58,50.66) | 9.54 (-33.72,52.79) | 10.86 (-34.44,56.16) | 11.49 (-33.44,56.42) | 12.61 (-32.43,57.65) | 13.24 (-31.88,58.36) | 13.87 (-31.01,58.75) | 20.26 (-26.10,66.62) | 20.42 (-24.74,65.58) | 21.11 (-24.05,66.27) | 21.93 (-19.62,63.48) | 23.55 (-21.34,68.44) | 26.34 (-14.57,67.25) |
| **-40.21 (-73.91,-6.52)** | **-31.89 (-59.74,-4.03)** | -12.66 (-47.15,21.82) | -3.80 (-31.28,23.68) | -5.54 (-50.66,39.58) | RC+ MOX +AI | 4.00 (-19.66,27.65) | 5.32 (-21.89,32.53) | 5.95 (-20.65,32.55) | 7.07 (-19.71,33.85) | 7.70 (-19.21,34.61) | 8.33 (-18.19,34.85) | 14.72 (-14.22,43.66) | 14.88 (-12.11,41.87) | 15.57 (-11.42,42.56) | 16.39 (-3.99,36.76) | 18.01 (-8.53,44.55) | 20.80 (1.77,39.83) |
| **-44.21 (-75.37,-13.06)** | **-35.88 (-60.61,-11.16)** | -16.66 (-48.67,15.35) | -7.80 (-32.10,16.51) | -9.54 (-52.79,33.72) | -4.00 (-27.65,19.66) | RC+ACU | 1.32 (-22.68,25.32) | 1.95 (-21.35,25.25) | 3.07 (-20.44,26.58) | 3.70 (-19.95,27.36) | 4.33 (-18.88,27.54) | 10.72 (-15.22,36.66) | 10.88 (-12.86,34.62) | 11.57 (-12.17,35.32) | 12.39 (-3.44,28.22) | 14.01 (-9.22,37.24) | 16.80 (2.75,30.86) |
| **-45.53 (-79.47,-11.60)** | **-37.21 (-65.35,-9.06)** | -17.98 (-52.70,16.74) | -9.12 (-36.90,18.66) | -10.86 (-56.16,34.44) | -5.32 (-32.53,21.89) | -1.32 (-25.32,22.68) | RC+WA+ACUP | 0.63 (-26.27,27.53) | 1.75 (-25.34,28.84) | 2.38 (-24.83,29.59) | 3.01 (-23.81,29.83) | 9.40 (-19.82,38.62) | 9.56 (-17.73,36.85) | 10.25 (-17.04,37.54) | 11.07 (-9.70,31.84) | 12.69 (-14.15,39.53) | 15.48 (-3.97,34.93) |
| **-46.16 (-79.61,-12.72)** | **-37.84 (-65.39,-10.28)** | -18.61 (-52.85,15.63) | -9.75 (-36.93,17.43) | -11.49 (-56.42,33.44) | -5.95 (-32.55,20.65) | -1.95 (-25.25,21.35) | -0.63 (-27.53,26.27) | RC+AA+PAT | 1.12 (-25.35,27.59) | 1.75 (-24.84,28.34) | 2.38 (-23.82,28.58) | 8.77 (-19.88,37.42) | 8.93 (-17.74,35.60) | 9.62 (-17.06,36.30) | 10.44 (-9.52,30.40) | 12.06 (-14.16,38.28) | 14.85 (-3.73,33.43) |
| **-47.28 (-80.88,-13.69)** | **-38.96 (-66.69,-11.22)** | -19.73 (-54.12,14.65) | -10.87 (-38.23,16.49) | -12.61 (-57.65,32.43) | -7.07 (-33.85,19.71) | -3.07 (-26.58,20.44) | -1.75 (-28.84,25.34) | -1.12 (-27.59,25.35) | RC+TEAS | 0.63 (-26.15,27.41) | 1.26 (-25.13,27.65) | 7.65 (-21.17,36.47) | 7.81 (-19.05,34.67) | 8.50 (-18.36,35.36) | 9.32 (-10.89,29.52) | 10.94 (-15.47,37.35) | 13.73 (-5.12,32.58) |
| **-47.91 (-81.61,-14.22)** | **-39.59 (-67.44,-11.73)** | -20.36 (-54.85,14.12) | -11.50 (-38.98,15.98) | -13.24 (-58.36,31.88) | -7.70 (-34.61,19.21) | -3.70 (-27.36,19.95) | -2.38 (-29.59,24.83) | -1.75 (-28.34,24.84) | -0.63 (-27.41,26.15) | RC+AA+AI | 0.63 (-25.89,27.15) | 7.02 (-21.92,35.96) | 7.18 (-19.80,34.16) | 7.87 (-19.12,34.86) | 8.69 (-11.68,29.06) | 10.31 (-16.22,36.84) | 13.10 (-5.93,32.13) |
| **-48.54 (-81.92,-15.16)** | **-40.22 (-67.69,-12.74)** | -20.99 (-55.17,13.19) | -12.13 (-39.23,14.97) | -13.87 (-58.75,31.01) | -8.33 (-34.85,18.19) | -4.33 (-27.54,18.88) | -3.01 (-29.83,23.81) | -2.38 (-28.58,23.82) | -1.26 (-27.65,25.13) | -0.63 (-27.15,25.89) | RC+ACU+PAT | 6.39 (-22.19,34.97) | 6.55 (-20.04,33.14) | 7.24 (-19.36,33.84) | 8.06 (-11.80,27.91) | 9.68 (-16.46,35.82) | 12.47 (-6.00,30.94) |
| **-54.93 (-90.27,-19.60)** | **-46.61 (-76.43,-16.79)** | -27.38 (-63.47,8.71) | -18.52 (-47.99,10.95) | -20.26 (-66.62,26.10) | -14.72 (-43.66,14.22) | -10.72 (-36.66,15.22) | -9.40 (-38.62,19.82) | -8.77 (-37.42,19.88) | -7.65 (-36.47,21.17) | -7.02 (-35.96,21.92) | -6.39 (-34.97,22.19) | RC+ACUP | 0.16 (-28.85,29.17) | 0.85 (-28.16,29.86) | 1.67 (-21.32,24.66) | 3.29 (-25.30,31.88) | 6.08 (-15.73,27.89) |
| **-55.09 (-88.85,-21.34)** | **-46.77 (-74.69,-18.84)** | -27.54 (-62.09,7.00) | -18.68 (-46.24,8.88) | -20.42 (-65.58,24.74) | -14.88 (-41.87,12.11) | -10.88 (-34.62,12.86) | -9.56 (-36.85,17.73) | -8.93 (-35.60,17.74) | -7.81 (-34.67,19.05) | -7.18 (-34.16,19.80) | -6.55 (-33.14,20.04) | -0.16 (-29.17,28.85) | RC+ACU+FT | 0.69 (-26.37,27.75) | 1.51 (-18.97,21.98) | 3.13 (-23.48,29.74) | 5.92 (-13.21,25.05) |
| **-55.78 (-89.54,-22.03)** | **-47.46 (-75.39,-19.53)** | -28.23 (-62.78,6.31) | -19.37 (-46.93,8.19) | -21.11 (-66.27,24.05) | -15.57 (-42.56,11.42) | -11.57 (-35.32,12.17) | -10.25 (-37.54,17.04) | -9.62 (-36.30,17.06) | -8.50 (-35.36,18.36) | -7.87 (-34.86,19.12) | -7.24 (-33.84,19.36) | -0.85 (-29.86,28.16) | -0.69 (-27.75,26.37) | RC+PAT | 0.82 (-19.66,21.30) | 2.44 (-24.17,29.05) | 5.23 (-13.91,24.37) |
| **-56.60 (-85.34,-27.86)** | **-48.27 (-69.88,-26.67)** | -29.05 (-58.72,0.62) | -20.19 (-41.31,0.94) | -21.93 (-63.48,19.62) | -16.39 (-36.76,3.99) | -12.39 (-28.22,3.44) | -11.07 (-31.84,9.70) | -10.44 (-30.40,9.52) | -9.32 (-29.52,10.89) | -8.69 (-29.06,11.68) | -8.06 (-27.91,11.80) | -1.67 (-24.66,21.32) | -1.51 (-21.98,18.97) | -0.82 (-21.30,19.66) | RC+AA | 1.62 (-18.25,21.50) | 4.41 (-2.87,11.69) |
| **-58.22 (-91.62,-24.83)** | **-49.90 (-77.39,-22.40)** | -30.67 (-64.86,3.52) | -21.81 (-48.93,5.31) | -23.55 (-68.44,21.34) | -18.01 (-44.55,8.53) | -14.01 (-37.24,9.22) | -12.69 (-39.53,14.15) | -12.06 (-38.28,14.16) | -10.94 (-37.35,15.47) | -10.31 (-36.84,16.22) | -9.68 (-35.82,16.46) | -3.29 (-31.88,25.30) | -3.13 (-29.74,23.48) | -2.44 (-29.05,24.17) | -1.62 (-21.50,18.25) | RC+AA+ACUP | 2.79 (-15.70,21.28) |
| **-61.01 (-88.82,-33.21)** | **-52.69 (-73.03,-32.34)** | **-33.46 (-62.22,-4.70)** | **-24.60 (-44.43,-4.77)** | -26.34 (-67.25,14.57) | **-20.80 (-39.83,-1.77)** | **-16.80 (-30.86,-2.75)** | -15.48 (-34.93,3.97) | -14.85 (-33.43,3.73) | -13.73 (-32.58,5.12) | -13.10 (-32.13,5.93) | -12.47 (-30.94,6.00) | -6.08 (-27.89,15.73) | -5.92 (-25.05,13.21) | -5.23 (-24.37,13.91) | -4.41 (-11.69,2.87) | -2.79 (-21.28,15.70) | RC |

Abbreviations: Mean difference and 95% credibility intervals between 18 interventions: indirect comparisons from network meta-analysis. Results are the MD and related 95% CIs in the row-defining treatment compared with the MD in the column-defining treatment. MD >0 favors the column-defining treatment and vice versa. The significant result is in bold and underlined. TFD: time to first defecation; RC: routine care; RC+MOX: routine care combined with moxibustion; RC+AA: routine care combined with auricular acupuncture; RC+ACUP: routine care combined with acupressure; RC+AA+ACUP: routine care combined with auricular acupuncture and acupressure; RC+ACU: routine care combined with acupuncture; RC+MOX+CUP: routine care combined with moxibustion and cupping; RC+MOX+AI: routine care combined with moxibustion and acupoint injection; RC+WA+ACUP: routine care combined with warming acupuncture and acupressure; RC+EA+PAT: routine care combined with electro-acupuncture and point application therapy; RC+AI: routine care combined with and acupoint injection; RC+AA+AI: routine care combined with auricular acupuncture and acupoint injection; RC+ACU+ FT: routine care combined with acupuncture and functional training; RC+ACU+PAT: routine care combined with acupuncture and point application therapy; RC+FBT: routine care combined with Foot bath therapy; RC+TEAS: routine care combined with transcutaneous electrical acupoint stimulation; RC+AA+PAT: routine care combined with auricular acupuncture and point application therapy; RC+PAT: routine care combined with point application therapy.

Supplementary Table S11. Head-to-head comparisons for an TFF of different interventions

| **RC+AI** | **RC+ACU** | **RC+MOX +AI** | **RC+ACUP** | **RC+EA+PAT** | **RC+WA+ACUP** | **RC+MOX** | **RC+AA +AI** | **RC+MOX+CUP** | **RC+TEAS** | **RC+ACU+PAT** | **RC+AA+PAT** | **RC+AA** | **RC+PAT** | **RC+ACU+FT** | **RC+AA+ACUP** | **RC** |
| --- | --- | --- | --- | --- | --- | --- | --- | --- | --- | --- | --- | --- | --- | --- | --- | --- |
| RC+AI | 7.55 (-10.29,25.40) | 7.50 (-13.99,28.99) | 9.15 (-10.33,28.63) | 7.84 (-18.45,34.13) | 10.08 (-11.53,31.69) | 13.70 (-3.28,30.68) | 14.70 (-9.72,39.12) | 15.42 (-6.19,37.03) | 17.36 (-3.79,38.51) | 18.20 (-2.85,39.25) | 19.48 (-2.84,41.80) | 19.66 (3.35,35.98) | 20.50 (-1.29,42.29) | 24.39 (3.10,45.68) | 25.59 (4.54,46.64) | 58.22 (24.83,91.62) |
| -7.55 (-25.40,10.29) | RC+ACU | -0.05 (-17.85,17.75) | 1.60 (-13.72,16.91) | 0.29 (-23.08,23.66) | 2.53 (-15.42,20.47) | 6.15 (-5.83,18.13) | 7.15 (-14.10,28.39) | 7.87 (-10.08,25.81) | 9.81 (-7.59,27.20) | 10.65 (-6.62,27.92) | 11.93 (-6.88,30.73) | 12.11 (1.10,23.12) | 12.95 (-5.22,31.12) | 16.84 (-0.72,34.39) | 18.04 (0.77,35.31) | 49.90 (22.40,77.39) |
| -7.50 (-28.99,13.99) | 0.05 (-17.75,17.85) | RC+MOX +AI | 1.65 (-17.79,21.09) | 0.34 (-25.92,26.60) | 2.58 (-18.99,24.15) | 6.20 (-10.73,23.14) | 7.20 (-17.18,31.58) | 7.92 (-13.65,29.49) | 9.86 (-11.26,30.98) | 10.70 (-10.32,31.72) | 11.98 (-10.31,34.27) | 12.16 (-4.10,28.43) | 13.00 (-8.76,34.76) | 16.89 (-4.36,38.14) | 18.09 (-2.92,39.10) | 30.67 (-3.52,64.86) |
| -9.15 (-28.63,10.33) | -1.60 (-16.91,13.72) | -1.65 (-21.09,17.79) | RC+ACUP | -1.31 (-25.95,23.33) | 0.93 (-18.64,20.51) | 4.55 (-9.75,18.86) | 5.55 (-17.08,28.19) | 6.27 (-13.30,25.84) | 8.21 (-10.86,27.28) | 9.05 (-9.90,28.01) | 10.33 (-10.03,30.69) | 10.52 (-2.99,24.02) | 11.35 (-8.43,31.13) | 15.24 (-3.98,34.46) | 16.44 (-2.51,35.40) | 21.81 (-5.31,48.93) |
| -7.84 (-34.13,18.45) | -0.29 (-23.66,23.08) | -0.34 (-26.60,25.92) | 1.31 (-23.33,25.95) | RC+EA+PAT | 2.24 (-24.12,28.60) | 5.86 (-16.86,28.58) | 6.86 (-21.84,35.56) | 7.58 (-18.78,33.94) | 9.52 (-16.46,35.50) | 10.36 (-15.54,36.26) | 11.64 (-15.31,38.59) | 11.82 (-10.40,34.05) | 12.66 (-13.85,39.17) | 16.55 (-9.54,42.64) | 17.75 (-8.15,43.65) | 23.55 (-21.34,68.44) |
| -10.08 (-31.69,11.53) | -2.53 (-20.47,15.42) | -2.58 (-24.15,18.99) | -0.93 (-20.51,18.64) | -2.24 (-28.60,24.12) | RC+WA+ACUP | 3.62 (-13.47,20.71) | 4.62 (-19.87,29.11) | 5.34 (-16.35,27.03) | 7.28 (-13.96,28.52) | 8.12 (-13.02,29.26) | 9.40 (-13.01,31.81) | 9.58 (-6.84,26.01) | 10.42 (-11.46,32.30) | 14.31 (-7.06,35.68) | 15.51 (-5.63,36.65) | 18.01 (-8.53,44.55) |
| -13.70 (-30.68,3.28) | -6.15 (-18.13,5.83) | -6.20 (-23.14,10.73) | -4.55 (-18.86,9.75) | -5.86 (-28.58,16.86) | -3.62 (-20.71,13.47) | RC+MOX | 1.00 (-19.53,21.52) | 1.72 (-15.37,18.81) | 3.66 (-12.85,20.16) | 4.50 (-11.88,20.88) | 5.78 (-12.21,23.76) | 5.96 (-3.59,15.52) | 6.80 (-10.53,24.12) | 10.69 (-5.99,27.37) | 11.89 (-4.49,28.26) | 14.01 (-9.22,37.24) |
| -14.70 (-39.12,9.72) | -7.15 (-28.39,14.10) | -7.20 (-31.58,17.18) | -5.55 (-28.19,17.08) | -6.86 (-35.56,21.84) | -4.62 (-29.11,19.87) | -1.00 (-21.52,19.53) | RC+AA +AI | 0.72 (-23.77,25.21) | 2.66 (-21.43,26.75) | 3.50 (-20.50,27.50) | 4.78 (-20.34,29.90) | 4.96 (-15.01,24.94) | 5.80 (-18.86,30.46) | 9.69 (-14.52,33.90) | 10.89 (-13.11,34.89) | 12.69 (-14.15,39.53) |
| -15.42 (-37.03,6.19) | -7.87 (-25.81,10.08) | -7.92 (-29.49,13.65) | -6.27 (-25.84,13.30) | -7.58 (-33.94,18.78) | -5.34 (-27.03,16.35) | -1.72 (-18.81,15.37) | -0.72 (-25.21,23.77) | RC+MOX+CUP | 1.94 (-19.30,23.18) | 2.78 (-18.36,23.92) | 4.06 (-18.35,26.47) | 4.24 (-12.18,20.67) | 5.08 (-16.80,26.96) | 8.97 (-12.40,30.34) | 10.17 (-10.96,31.30) | 12.06 (-14.16,38.28) |
| -17.36 (-38.51,3.79) | -9.81 (-27.20,7.59) | -9.86 (-30.98,11.26) | -8.21 (-27.28,10.86) | -9.52 (-35.50,16.46) | -7.28 (-28.52,13.96) | -3.66 (-20.16,12.85) | -2.66 (-26.75,21.43) | -1.94 (-23.18,19.30) | RC+TEAS | 0.84 (-19.83,21.51) | 2.12 (-19.85,24.09) | 2.30 (-13.51,18.12) | 3.14 (-18.29,24.57) | 7.03 (-13.88,27.94) | 8.23 (-12.44,28.90) | 10.94 (-15.47,37.35) |
| -18.20 (-39.25,2.85) | -10.65 (-27.92,6.62) | -10.70 (-31.72,10.32) | -9.05 (-28.01,9.90) | -10.36 (-36.26,15.54) | -8.12 (-29.26,13.02) | -4.50 (-20.88,11.88) | -3.50 (-27.50,20.50) | -2.78 (-23.92,18.36) | -0.84 (-21.51,19.83) | RC+ACU+PAT | 1.28 (-20.59,23.15) | 1.46 (-14.22,17.15) | 2.30 (-19.03,23.63) | 6.19 (-14.62,27.00) | 7.39 (-13.18,27.96) | 10.31 (-16.22,36.84) |
| -19.48 (-41.80,2.84) | -11.93 (-30.73,6.88) | -11.98 (-34.27,10.31) | -10.33 (-30.69,10.03) | -11.64 (-38.59,15.31) | -9.40 (-31.81,13.01) | -5.78 (-23.76,12.21) | -4.78 (-29.90,20.34) | -4.06 (-26.47,18.35) | -2.12 (-24.09,19.85) | -1.28 (-23.15,20.59) | RC+AA+PAT | 0.18 (-17.17,17.54) | 1.02 (-21.57,23.61) | 4.91 (-17.19,27.01) | 6.11 (-15.76,27.98) | 9.68 (-16.46,35.82) |
| **-19.66 (-35.98,-3.35)** | -12.11 (-23.12,-1.10) | -12.16 (-28.43,4.10) | -10.52 (-24.02,2.99) | -11.82 (-34.05,10.40) | -9.58 (-26.01,6.84) | -5.96 (-15.52,3.59) | -4.96 (-24.94,15.01) | -4.24 (-20.67,12.18) | -2.30 (-18.12,13.51) | -1.46 (-17.15,14.22) | -0.18 (-17.54,17.17) | RC+AA | 0.84 (-15.83,17.51) | 4.73 (-11.27,20.72) | 5.93 (-9.76,21.61) | 3.29 (-25.30,31.88) |
| -20.50 (-42.29,1.29) | **-12.95 (-31.12,5.22)** | -13.00 (-34.76,8.76) | -11.35 (-31.13,8.43) | -12.66 (-39.17,13.85) | -10.42 (-32.30,11.46) | -6.80 (-24.12,10.53) | -5.80 (-30.46,18.86) | -5.08 (-26.96,16.80) | -3.14 (-24.57,18.29) | -2.30 (-23.63,19.03) | -1.02 (-23.61,21.57) | -0.84 (-17.51,15.83) | RC+PAT | 3.89 (-17.67,25.45) | 5.09 (-16.24,26.42) | 3.13 (-23.48,29.74) |
| **-24.39 (-45.68,-3.10)** | -16.84 (-34.39,0.72) | -16.89 (-38.14,4.36) | -15.24 (-34.46,3.98) | -16.55 (-42.64,9.54) | -14.31 (-35.68,7.06) | -10.69 (-27.37,5.99) | -9.69 (-33.90,14.52) | -8.97 (-30.34,12.40) | -7.03 (-27.94,13.88) | -6.19 (-27.00,14.62) | -4.91 (-27.01,17.19) | -4.73 (-20.72,11.27) | -3.89 (-25.45,17.67) | RC+ACU+FT | 1.20 (-19.61,22.01) | 2.44 (-24.17,29.05) |
| **-25.59 (-46.64,-4.54)** | **-18.04 (-35.31,-0.77)** | -18.09 (-39.10,2.92) | -16.44 (-35.40,2.51) | -17.75 (-43.65,8.15) | -15.51 (-36.65,5.63) | -11.89 (-28.26,4.49) | -10.89 (-34.89,13.11) | -10.17 (-31.30,10.96) | -8.23 (-28.90,12.44) | -7.39 (-27.96,13.18) | -6.11 (-27.98,15.76) | -5.93 (-21.61,9.76) | -5.09 (-26.42,16.24) | -1.20 (-22.01,19.61) | RC+AA+ACUP | 1.62 (-18.25,21.50) |
| **-28.00 (-43.22,-12.78)** | **-20.45 (-29.76,-11.13)** | **-20.50 (-35.67,-5.33)** | **-18.85 (-31.01,-6.69)** | -20.16 (-41.59,1.27) | **-17.92 (-33.26,-2.58)** | **-14.30 (-21.83,-6.76)** | -13.30 (-32.39,5.79) | -12.58 (-27.92,2.76) | -10.64 (-25.33,4.05) | -9.80 (-24.34,4.74) | -8.52 (-24.85,7.81) | **-8.34 (-14.21,-2.46)** | -7.50 (-23.10,8.10) | -3.61 (-18.49,11.27) | -2.41 (-16.95,12.13) | RC |

Abbreviations: Mean difference and 95% credibility intervals between 17 interventions: indirect comparisons from network meta-analysis. Results are the MD and related 95% CIs in the row-defining treatment compared with the MD in the column-defining treatment. MD >0 favors the column-defining treatment and vice versa. The significant result is in bold and underlined. TFF: Time to first flatus; RC: routine care; RC+MOX: routine care combined with moxibustion; RC+AA: routine care combined with auricular acupuncture; RC+ACUP: routine care combined with acupressure; RC+AA+ACUP: routine care combined with auricular acupuncture and acupressure; RC+ACU: routine care combined with acupuncture; RC+MOX+CUP: routine care combined with moxibustion and cupping; RC+MOX+AI: routine care combined with moxibustion and acupoint injection; RC+WA+ACUP: routine care combined with warming acupuncture and acupressure; RC+EA+PAT: routine care combined with electro-acupuncture and point application therapy; RC+AI: routine care combined with and acupoint injection; RC+AA+AI: routine care combined with auricular acupuncture and acupoint injection; RC+ACU+FT: routine care combined with acupuncture and functional training; RC+ACU+PAT: routine care combined with acupuncture and point application therapy; RC+TEAS: routine care combined with transcutaneous electrical acupoint stimulation; RC+AA+PAT: routine care combined with auricular acupuncture and point application therapy; RC+PAT: routine care combined with point application therapy.

Supplementary Table S12. Evidence Quality of TFBS Score

| **Comparison** | | **Direct evidence** | **Indirect evidence** | **Network meta-analysis** |
| --- | --- | --- | --- | --- |
| RC+ACU | RC+MOX+AI | Low | Low | Low |
| RC+ACU | RC+ACUP | Moderate | Low | Moderate |
| RC+ACU | RC+AI+AA | - | Low | Low |
| RC+ACU | RC+AI | - | Very low | Very low |
| RC+ACU | RC+WA+ACUP | - | Low | Low |
| RC+ACU | RC+ACU +PAT | - | Low | Low |
| RC+ACU | RC+MOX+CUP | - | Low | Low |
| RC+ACU | RC+TEAS | - | Low | Low |
| RC+ACU | RC+AA | - | Low | Low |
| RC+ACU | RC+MOX | - | Very Low | Low |
| RC+ACU | RC+AA+ACUP | - | Low | Low |
| RC+ACU | RC+EA+PAT | - | Low | Low |
| RC+ACU | RC | - | Low | Low |
| RC+MOX+AI | RC+ACUP | - | Low | Low |
| RC+MOX+AI | RC+AI+AA | - | Low | Low |
| RC+MOX+AI | RC+AI | - | Low | Low |
| RC+MOX+AI | RC+WA+ACUP | - | Low | Low |
| RC+MOX+AI | RC+PAT+ACU | Moderate | Moderate | Moderate |
| RC+MOX+AI | RC+MOX+CUP | - | Low | Low |
| RC+MOX+AI | RC+TEAS | - | Low | Low |
| RC+MOX+AI | RC+AA | - | Low | Low |
| RC+MOX+AI | RC+MOX | - | Very Low | Low |
| RC+MOX+AI | RC+AA+ACUP | - | Low | Low |
| RC+MOX+AI | RC+EA+PAT | - | Low | Low |
| RC+MOX+AI | RC | - | Low | Low |
| RC+ACUP | RC+AI+AA | - | Very Low | Very Low |
| RC+ACUP | RC+AI | - | Low | Low |
| RC+ACUP | RC+WA+ACUP | - | Low | Low |
| RC+ACUP | RC+PAT+ACU | - | Low | Low |
| RC+ACUP | RC+MOX+CUP | - | Low | Low |
| RC+ACUP | RC+TEAS | Low | Low | Low |
| RC+ACUP | RC+AA | - | Low | Low |
| RC+ACUP | RC+MOX | - | Low | Low |
| RC+ACUP | RC+AA+ACUP | - | - | - |
| RC+ACUP | RC+EA+PAT | - | Low | Low |
| RC+ACUP | RC | - | Low | Low |
| RC+AI+AA | RC+AI | - | Low | Low |
| RC+AI+AA | RC+WA+ACUP | - | Low | Low |
| RC+AI+AA | RC+PAT+ACU | - | Very Low | Very Low |
| RC+AI+AA | RC+MOX+CUP | Moderate | Low | Low |
| RC+AI+AA | RC+TEAS | - | Low | Low |
| RC+AI+AA | RC+AA | Low | Low | Low |
| RC+AI+AA | RC+MOX | - | Low | Low |
| RC+AI+AA | RC+AA+ACUP | Low | Low | Low |
| RC+AI+AA | RC+EA+PAT | - | Low | Low |
| RC+AI+AA | RC | - | Low | Low |
| RC+AI | RC+WA+ACUP | - | Low | Low |
| RC+AI | RC+PAT+ACU | - | Low | Low |
| RC+AI | RC+MOX+CUP | - | Low | Low |
| RC+AI | RC+TEAS | - | Low | Low |
| RC+AI | RC+AA | - | Low | Low |
| RC+AI | RC+MOX | - | Low | Low |
| RC+AI | RC+AA+ACUP | - | Low | Low |
| RC+AI | RC+EA+PAT | - | Low | Low |
| RC+AI | RC | - | Low | Low |
| RC+WA+ACUP | RC+PAT+ACU | - | Low | Low |
| RC+WA+ACUP | RC+MOX+CUP | - | Low | Low |
| RC+WA+ACUP | RC+TEAS | - | Low | Low |
| RC+WA+ACUP | RC+AA | - | - | - |
| RC+WA+ACUP | RC+MOX | - | Low | Low |
| RC+WA+ACUP | RC+AA+ACUP | - | Low | Low |
| RC+WA+ACUP | RC+EA+PAT | - | Low | Low |
| RC+WA+ACUP | RC | - | Low | Low |
| RC+PAT+ACU | RC+MOX+CUP | - | Low | Low |
| RC+PAT+ACU | RC+TEAS | - | Low | Low |
| RC+PAT+ACU | RC+AA | - | Low | Low |
| RC+PAT+ACU | RC+MOX | - | Low | Low |
| RC+PAT+ACU | RC+AA+ACUP | - | Low | Low |
| RC+PAT+ACU | RC+EA+PAT | - | Low | Low |
| RC+PAT+ACU | RC | - | Low | Low |
| RC+MOX+CUP | RC+TEAS | - | Low | Low |
| RC+MOX+CUP | RC+AA | Moderate | Low | Low |
| RC+MOX+CUP | RC+MOX | - | Low | Low |
| RC+MOX+CUP | RC+AA+ACUP | - | Low | Low |
| RC+MOX+CUP | RC+EA+PAT | - | Low | Low |
| RC+MOX+CUP | RC | - | Low | Low |
| RC+TEAS | RC+AA | - | Very Low | Very Low |
| RC+TEAS | RC+MOX | - | Low | Low |
| RC+TEAS | RC+AA+ACUP | - | Low | Low |
| RC+TEAS | RC+EA+PAT | Low | Low | Low |
| RC+TEAS | RC | - | Low | Low |
| RC+AA | RC+MOX | - | Low | Low |
| RC+AA | RC+AA+ACUP | - | Low | Low |
| RC+AA | RC+EA+PAT | - | Low | Low |
| RC+AA | RC | Very low | Low | Very low |
| RC+MOX | RC+AA+ACUP | - | Low | Low |
| RC+MOX | RC+EA+PAT | - | Low | Low |
| RC+MOX | RC | - | Low | Low |
| RC+AA+ACUP | RC+EA+PAT | - | Low | Low |
| RC+AA+ACUP | RC | - | Low | Low |
| RC+EA+PAT | RC | - | Low | Low |

Abbreviations :TFBS: Time of first bowel sounds; RC: routine care; RC+MOX: routine care combined with moxibustion; RC+AA: routine care combined with auricular acupuncture; RC+ACUP: routine care combined with acupressure; RC+TEAS: routine care combined with transcutaneous electrical acupoint stimulation; RC+AA+ACUP: routine care combined with auricular acupuncture and acupressure; RC+ACU: routine care combined with acupuncture; RC+MOX+CUP: routine care combined with moxibustion and cupping; RC+MOX+AI: routine care combined with moxibustion and acupoint injection; RC+WA+ACUP: routine care combined with warming acupuncture and acupressure; RC+EA+PAT: routine care combined with electro-acupuncture and point application therapy; RC+AI: routine care combined with and acupoint injection; RC+AA+AI: routine care combined with auricular acupuncture and acupoint injection; RC+ACU+PAT: routine care combined with acupuncture and point application therapy.

Supplementary Table S13. Evidence Quality of TFD

| **Comparison** | | **Direct evidence** | **Indirect evidence** | **Network meta-analysis** |
| --- | --- | --- | --- | --- |
| RC+MOX+CUP | RC+MOX | - | Low | Low |
| RC+MOX+CUP | RC+FBT | Moderate | Moderate | Moderate |
| RC+MOX+CUP | RC+AI | - | Low | Low |
| RC+MOX+CUP | RC+EA+PAT | - | low | Very low |
| RC+MOX+CUP | RC+MOX+AI | - | Very low | Very low |
| RC+MOX+CUP | RC+ACU | - | Low | Low |
| RC+MOX+CUP | RC+WA+ACUP | - | Low | Low |
| RC+MOX+CUP | RC+AA+PAT | - | Low | Low |
| RC+MOX+CUP | RC+TEAS | - | Low | Low |
| RC+MOX+CUP | RC+AA+AI | - | Low | Low |
| RC+MOX+CUP | RC+ACU+PAT | Low | Low | Low |
| RC+MOX+CUP | RC+ACUP | - | Low | Low |
| RC+MOX+CUP | RC+ACU+FT | - | Very low | Very low |
| RC+MOX+CUP | RC+PAT | - | Low | Low |
| RC+MOX+CUP | RC+AA | Low | Low | Low |
| RC+MOX+CUP | RC+AA+ACUP | - | Low | Low |
| RC+MOX+CUP | RC | - | Low | Low |
| RC+MOX | RC+EA+PAT | Low | Low | Low |
| RC+MOX | RC+MOX+AI | Low | Low | Low |
| RC+MOX | RC+ACU | - | Low | Low |
| RC+MOX | RC+WA+ACUP | - | Very Low | Very Low |
| RC+MOX | RC+AA+PAT | - | Low | Low |
| RC+MOX | RC+TEAS | - | Low | Low |
| RC+MOX | RC+AA+AI | - | Low | Low |
| RC+MOX | RC+ACU+PAT | - | Low | Low |
| RC+MOX | RC+ACUP | - | Low | Low |
| RC+MOX | RC+ACU+FT | Low | Low | Low |
| RC+MOX | RC+PAT | - | Low | Low |
| RC+MOX | RC+AA | - | Low | Low |
| RC+MOX | RC+AA+ACUP | - | Low | Low |
| RC+MOX | RC | - | Low | Low |
| RC+FBT | RC+AI | Low | Low | Low |
| RC+FBT | RC+EA+PAT | - | Low | Low |
| RC+FBT | RC+MOX+AI | - | Low | Low |
| RC+FBT | RC+ACU | - | Low | Low |
| RC+FBT | RC+WA+ACUP | - | Low | Low |
| RC+FBT | RC+AA+PAT | - | Low | Low |
| RC+FBT | RC+TEAS | - | Low | Low |
| RC+FBT | RC+AA+AI | Low | Low | Low |
| RC+FBT | RC+ACU+PAT | - | Low | Low |
| RC+FBT | RC+ACUP | - | Low | Low |
| RC+FBT | RC+ACU+FT | - | Low | Low |
| RC+FBT | RC+PAT | - | Low | Low |
| RC+FBT | RC+AA | Low | Low | Low |
| RC+FBT | RC+AA+ACUP | - | Low | Low |
| RC+FBT | RC | - | Low | Low |
| RC+AI | RC+EA+PAT | - | Low | Low |
| RC+AI | RC+MOX+AI | - | Low | Low |
| RC+AI | RC+ACU | Low | Low | Low |
| RC+AI | RC+WA+ACUP | - | Low | Low |
| RC+AI | RC+AA+PAT | - | Low | Low |
| RC+AI | RC+TEAS | - | Low | Low |
| RC+AI | RC+AA+AI | - | Low | Low |
| RC+AI | RC+ACU+PAT | - | Low | Low |
| RC+AI | RC+ACUP | - | Low | Low |
| RC+AI | RC+ACU+FT | - | Low | Low |
| RC+AI | RC+PAT | - | Low | Low |
| RC+AI | RC+AA | - | Low | Low |
| RC+AI | RC+AA+ACUP | - | Low | Low |
| RC+AI | RC | Moderate | Low | Low |
| RC+EA+PAT | RC+MOX+AI | - | Low | Low |
| RC+EA+PAT | RC+ACU | - | Low | Low |
| RC+EA+PAT | RC+WA+ACUP | - | Low | Low |
| RC+EA+PAT | RC+AA+PAT | - | Low | Low |
| RC+EA+PAT | RC+TEAS | - | Low | Low |
| RC+EA+PAT | RC+AA+AI | Low | Low | Low |
| RC+EA+PAT | RC+ACU+PAT | - | Low | Low |
| RC+EA+PAT | RC+ACUP | - | Low | Low |
| RC+EA+PAT | RC+ACU+FT | - | Low | Low |
| RC+EA+PAT | RC+PAT | - | Low | Low |
| RC+EA+PAT | RC+AA | Low | Low | Low |
| RC+EA+PAT | RC | - | Low | Low |
| RC+MOX+AI | RC+ACU | - | Low | Low |
| RC+MOX+AI | RC+WA+ACUP | - | Low | Low |
| RC+MOX+AI | RC+AA+PAT | Low | Low | Low |
| RC+MOX+AI | RC+TEAS | - | Low | Low |
| RC+MOX+AI | RC+AA+AI | - | Low | Low |
| RC+MOX+AI | RC+ACU+PAT | - | Low | Low |
| RC+MOX+AI | RC+ACUP | - | Low | Low |
| RC+MOX+AI | RC+ACU+FT | Low | Low | Low |
| RC+MOX+AI | RC+PAT | - | Low | Low |
| RC+MOX+AI | RC+AA | - | - | - |
| RC+MOX+AI | RC | - | Low | Low |
| RC+ACU | RC+MOX+AI | Low | Low | Low |
| RC+ACU | RC+WA+ACUP | - | Low | Low |
| RC+ACU | RC+AA+PAT | - | Low | Low |
| RC+ACU | RC+TEAS | - | Low | Low |
| RC+ACU | RC+AA+AI | - | Low | Low |
| RC+ACU | RC+ACU+PAT | Low | Low | Low |
| RC+ACU | RC+ACUP | - | Low | Low |
| RC+ACU | RC+ACU+FT | - | Low | Low |
| RC+ACU | RC+PAT | - | Low | Low |
| RC+ACU | RC+AA | Low | Low | Low |
| RC+ACU | RC | - | Low | Low |
| RC+WA+ACUP | RC+AA+PAT | - | Low | Low |
| RC+WA+ACUP | RC+TEAS | - | Low | Low |
| RC+WA+ACUP | RC+AA+AI | - | Low | Low |
| RC+WA+ACUP | RC+ACU+PAT | Low | Low | Low |
| RC+WA+ACUP | RC+ACUP | - | Low | Low |
| RC+WA+ACUP | RC+ACU+FT | - | Low | Low |
| RC+WA+ACUP | RC+PAT | - | Low | Low |
| RC+WA+ACUP | RC+AA | Low | Low | Low |
| RC+WA+ACUP | RC | - | Low | Low |
| RC+AA+PAT | RC+TEAS | - | Low | Low |
| RC+AA+PAT | RC+AA+AI | - | Low | Low |
| RC+AA+PAT | RC+ACU+PAT | - | Low | Low |
| RC+AA+PAT | RC+ACUP | Low | Low | Low |
| RC+AA+PAT | RC+ACU+FT | - | Low | Low |
| RC+AA+PAT | RC+PAT | - | Low | Low |
| RC+AA+PAT | RC+AA | - | Low | Low |
| RC+AA+PAT | RC | Low | Low | Low |
| RC+TEAS | RC+AA+AI | - | Low | Low |
| RC+TEAS | RC+ACU+PAT | - | Low | Low |
| RC+TEAS | RC+ACUP | - | Low | Low |
| RC+TEAS | RC+ACU+FT | - | Low | Low |
| RC+TEAS | RC+PAT | Low | Low | Low |
| RC+TEAS | RC+AA | - | Low | Low |
| RC+TEAS | RC | - | Low | Low |
| RC+AA+AI | RC+ACU+PAT | - | Low | Low |
| RC+AA+AI | RC+ACUP | Low | Low | Low |
| RC+AA+AI | RC+ACU+FT | - | Low | Low |
| RC+AA+AI | RC+PAT | - | Low | Low |
| RC+AA+AI | RC+AA | - | Low | Low |
| RC+AA+AI | RC | - | Low | Low |
| RC+ACU+PAT | RC+ACUP | Low | Low | Low |
| RC+ACU+PAT | RC+ACU+FT | - | Low | Low |
| RC+ACU+PAT | RC+PAT | - | Low | Low |
| RC+ACU+PAT | RC+AA | - | Low | Low |
| RC+ACU+PAT | RC | Low | Low | Low |
| RC+ACUP | RC+ACU+FT | - | Low | Low |
| RC+ACUP | RC+PAT | - | Low | Low |
| RC+ACUP | RC+AA | - | Low | Low |
| RC+ACUP | RC | - | Low | Low |
| RC+ACU+FT | RC+PAT | Low | Low | Low |
| RC+ACU+FT | RC+AA | - | Low | Low |
| RC+ACU+FT | RC | - | Low | Low |
| RC+PAT | RC+AA | - | Low | Low |
| RC+PAT | RC | Low | Low | Low |
| RC+AA | RC | - | Low | Low |
| RC+AA+ACUP | RC | - | Low | Low |

Abbreviations: TFD: time to first defecation; RC: routine care; RC+MOX: routine care combined with moxibustion; RC+AA: routine care combined with auricular acupuncture; RC+ACUP: routine care combined with acupressure; RC+AA+ACUP: routine care combined with auricular acupuncture and acupressure; RC+ACU: routine care combined with acupuncture; RC+MOX+CUP: routine care combined with moxibustion and cupping; RC+MOX+AI: routine care combined with moxibustion and acupoint injection; RC+WA+ACUP: routine care combined with warming acupuncture and acupressure; RC+EA+PAT: routine care combined with electro-acupuncture and point application therapy; RC+AI: routine care combined with and acupoint injection; RC+AA+AI: routine care combined with auricular acupuncture and acupoint injection; RC+ACU+FT: routine care combined with acupuncture and functional training; RC+ACU+PAT: routine care combined with acupuncture and point application therapy; RC+FBT: routine care combined with Foot bath therapy; RC+TEAS: routine care combined with transcutaneous electrical acupoint stimulation; RC+AA+PAT: routine care combined with auricular acupuncture and point application therapy; RC+PAT: routine care combined with point application therapy.

Supplementary Table S14. Evidence Quality of TFF

| **Comparison** | | **Direct evidence** | **Indirect evidence** | **Network meta-analysis** |
| --- | --- | --- | --- | --- |
| RC+AI | RC+ACU | - | Low | Low |
| RC+AI | RC+MOX+AI | Moderate | Moderate | Moderate |
| RC+AI | RC+ACUP | - | Low | Low |
| RC+AI | RC+EA+PAT | - | low | Very low |
| RC+AI | RC+WA+ACUP | - | Very low | Very low |
| RC+AI | RC+MOX | - | Low | Low |
| RC+AI | RC+AA+AI | - | Low | Low |
| RC+AI | RC+MOX+CUP | - | Low | Low |
| RC+AI | RC+TEAS | - | Low | Low |
| RC+AI | RC+ACU+PAT | - | Low | Low |
| RC+AI | RC+AA | Low | Low | Low |
| RC+AI | RC+PAT | - | Low | Low |
| RC+AI | RC+AA+ACUP | - | Very low | Very low |
| RC+AI | RC | - | Low | Low |
| RC+ACU | RC+MOX+AI | Low | Low | Low |
| RC+ACU | RC+ACUP | Low | Low | Low |
| RC+ACU | RC+EA+PAT | - | Low | Low |
| RC+ACU | RC+WA+ACUP | - | Very Low | Very Low |
| RC+ACU | RC+MOX | - | Low | Low |
| RC+ACU | RC+AA+AI | - | Low | Low |
| RC+ACU | RC+MOX+CUP | - | Low | Low |
| RC+ACU | RC+TEAS | - | Low | Low |
| RC+ACU | RC+ACU+PAT | - | Low | Low |
| RC+ACU | RC+AA | Low | Low | Low |
| RC+ACU | RC+PAT | - | Low | Low |
| RC+ACU | RC+AA+ACUP | - | Low | Low |
| RC+ACU | RC | - | Low | Low |
| RC+MOX+AI | RC+ACUP | - | Low | Low |
| RC+MOX+AI | RC+EA+PAT | Low | Low | Low |
| RC+MOX+AI | RC+WA+ACUP | - | Low | Low |
| RC+MOX+AI | RC+MOX | - | Low | Low |
| RC+MOX+AI | RC+AA+AI | - | Low | Low |
| RC+MOX+AI | RC+MOX+CUP | - | Low | Low |
| RC+MOX+AI | RC+TEAS | - | Low | Low |
| RC+MOX+AI | RC+ACU+PAT | - | Low | Low |
| RC+MOX+AI | RC+AA | Low | Low | Low |
| RC+MOX+AI | RC+PAT | - | Low | Low |
| RC+MOX+AI | RC+AA+ACUP | - | Low | Low |
| RC+MOX+AI | RC | - | Low | Low |
| RC+ACUP | RC+EA+PAT | - | Low | Low |
| RC+ACUP | RC+WA+ACUP | - | Low | Low |
| RC+ACUP | RC+MOX | Low | Low | Low |
| RC+ACUP | RC+AA+AI | - | Low | Low |
| RC+ACUP | RC+MOX+CUP | - | Low | Low |
| RC+ACUP | RC+TEAS | - | Low | Low |
| RC+ACUP | RC+ACU+PAT | - | Low | Low |
| RC+ACUP | RC+AA | - | Low | Low |
| RC+ACUP | RC+PAT | - | Low | Low |
| RC+ACUP | RC+AA+ACUP | - | Low | Low |
| RC+ACUP | RC | - | Low | Low |
| RC+EA+PAT | RC+WA+ACUP | - | Low | Low |
| RC+EA+PAT | RC+MOX | - | Low | Low |
| RC+EA+PAT | RC+AA+AI | - | Low | Low |
| RC+EA+PAT | RC+MOX+CUP | - | Low | Low |
| RC+EA+PAT | RC+TEAS | - | Low | Low |
| RC+EA+PAT | RC+ACU+PAT | - | Low | Low |
| RC+EA+PAT | RC+AA | - | Low | Low |
| RC+EA+PAT | RC+PAT | - | Low | Low |
| RC+EA+PAT | RC+AA+ACUP | - | Low | Low |
| RC+EA+PAT | RC | - | Low | Low |
| RC+WA+ACUP | RC+MOX | - | Low | Low |
| RC+WA+ACUP | RC+AA+AI | - | Low | Low |
| RC+WA+ACUP | RC+MOX+CUP | - | Low | Low |
| RC+WA+ACUP | RC+TEAS | - | Low | Low |
| RC+WA+ACUP | RC+ACU+PAT | - | Low | Low |
| RC+WA+ACUP | RC+AA | - | Low | Low |
| RC+WA+ACUP | RC+PAT | - | Low | Low |
| RC+WA+ACUP | RC+AA+ACUP | - | Low | Low |
| RC+WA+ACUP | RC | - | Low | Low |
| RC+MOX | RC+AA+AI | - | Low | Low |
| RC+MOX | RC+MOX+CUP | - | Low | Low |
| RC+MOX | RC+TEAS | - | Low | Low |
| RC+MOX | RC+ACU+PAT | - | Low | Low |
| RC+MOX | RC+AA | - | Low | Low |
| RC+MOX | RC+PAT | - | Low | Low |
| RC+MOX | RC+AA+ACUP | - | Low | Low |
| RC+MOX | RC | - | Low | Low |
| RC+MOX | RC+MOX+CUP | - | Low | Low |
| RC+MOX | RC+TEAS | - | Low | Low |
| RC+AA+AI | RC+ACU+PAT | - | Low | Low |
| RC+AA+AI | RC+AA | - | Low | Low |
| RC+AA+AI | RC+PAT | - | Low | Low |
| RC+AA+AI | RC+AA+ACUP | - | Low | Low |
| RC+AA+AI | RC | - | Low | Low |
| RC+MOX+CUP | RC+TEAS | - | Low | Low |
| RC+MOX+CUP | RC+ACU+PAT | - | Low | Low |
| RC+MOX+CUP | RC+AA | - | Low | Low |
| RC+MOX+CUP | RC+PAT | - | Low | Low |
| RC+MOX+CUP | RC+AA+ACUP | - | Low | Low |
| RC+MOX+CUP | RC | - | Low | Low |
| RC+TEAS | RC+ACU+PAT | - | Low | Low |
| RC+TEAS | RC+AA | - | Low | Low |
| RC+TEAS | RC+PAT | - | Low | Low |
| RC+TEAS | RC+AA+ACUP | - | Low | Low |
| RC+TEAS | RC | - | Low | Low |
| RC+ACU+PAT | RC+AA | - | Low | Low |
| RC+ACU+PAT | RC+PAT | - | Low | Low |
| RC+ACU+PAT | RC+AA+ACUP | - | Low | Low |
| RC+ACU+PAT | RC | - | Low | Low |
| RC+AA | RC+PAT | - | Low | Low |
| RC+AA | RC+AA+ACUP | - | Low | Low |
| RC+AA | RC | - | Low | Low |
| RC+AA+ACUP | RC | - | Low | Low |

Abbreviations: TFF: Time to first flatus; RC: routine care; RC+MOX: routine care combined with moxibustion; RC+AA: routine care combined with auricular acupuncture; RC+ACUP: routine care combined with acupressure; RC+AA+ACUP: routine care combined with auricular acupuncture and acupressure; RC+ACU: routine care combined with acupuncture; RC+MOX+CUP: routine care combined with moxibustion and cupping; RC+MOX+AI: routine care combined with moxibustion and acupoint injection; RC+WA+ACUP: routine care combined with warming acupuncture and acupressure; RC+EA+PAT: routine care combined with electro-acupuncture and point application therapy; RC+AI: routine care combined with and acupoint injection; RC+AA+AI: routine care combined with auricular acupuncture and acupoint injection; RC+ACU+FT: routine care combined with acupuncture and functional training; RC+ACU+PAT: routine care combined with acupuncture and point application therapy; RC+TEAS: routine care combined with transcutaneous electrical acupoint stimulation; RC+AA+PAT: routine care combined with auricular acupuncture and point application therapy; RC+PAT: routine care combined with point application therapy.

Supplementary [S15| STATA 15.1 graphic code and official website link in this study](#_Toc119062189)

**Graphical code are as follows:**

Figure2. Network map for total score:

1. network setup mean sd n, studyvar(study) trtvar(trt)
2. network map

Figure 5 Funnel plot on publication bias

1. network convert pairs
2. netfunnel _y _stderr _t1 _t2 , random bycomp add(lfit _stderr _ES_CEN) noalpha

Figure 4. SUCRA for total score

1. network convert augment
2. set matsize 11000
3. network meta i, force
4. network forest
5. The next code operations need to be viewed in terms of the types of interventions corresponding to the different outcome indicators (TFBS, TFD, TFF)

For example:

- 1. SUCRA of TFBS: intervalplot, eform pred null (1) lab (A B C D E F G H I J K L M N) textsize(50)
  2. SUCRA of TFD: intervalplot, eform pred null (1) lab (A B C D E F G H I J K L M N O P Q) textsize(50)
  3. SUCRA of TFF: intervalplot, eform pred null (1) lab (A B C D E F G H I J K L M N O P Q R) textsize(50)

1. network rank min, zero all reps (10000) gen(prob)
2. sucra prob*, labels (A B C D E F) rankog

For example:

- 1. SUCRA of TFBS：sucra prob*, labels (A B C D E F G H I J K L M N) rankog
  2. SUCRA of TFD: sucra prob*, labels (A B C D E F G H I J K L M N O P Q) rankog
  3. SUCRA of TFF: sucra prob*, labels (A B C D E F G H I J K L M N O P Q R) rankog

1. sucra prob*, labels (A B C D E F G H I) lcol(blue)

For example:

- 1. SUCRA of TFBS：labels (A B C D E F G H I J K L M N) lcol(blue)
  2. SUCRA of TFD: labels (A B C D E F G H I J K L M N O P Q) lcol(blue)
  3. SUCRA of TFF: labels (A B C D E F G H I J K L M N O P Q R) lcol(blue)

Supplementary Table S12-S14| Head-to-head comparisons for TFBS/TFF/TFD of the different interventions

1. db netleague

**Official website link of STATA15.1:** **https://download.stata.com/download/**
